# High-Uniformity Planar Mini-Chip-Scale Packaged LEDs with Quantum Dot Converter for White Light Source

**DOI:** 10.1186/s11671-019-2993-z

**Published:** 2019-05-29

**Authors:** Lung-Chien Chen, Ching-Ho Tien, De-Fu Chen, Zhi-Ting Ye, Hao-Chung Kuo

**Affiliations:** 1 Department of Electro-optical Engineering, National Taipei University of Technology, No. 1, 3 Sec., Chung-Hsiao E. Rd, Taipei, 10608 Taiwan; 2 Department of Electro-Optical Engineering, National United University, 2, Lienda, Miaoli, 26063 Taiwan; 3 Department of Photonics and Institute of Electro-Optical Engineering, National Chiao Tung University, Hsinchu, 30010 Taiwan

**Keywords:** Mini-CSPLED, Quantum dot, GaN, Backlight

## Abstract

This study proposes a novel direct-lit mini-chip-scale packaged light-emitting diode (mini-CSPLED) backlight unit (BLU) that used quantum dot (QD) film, diffusion plate, and two prism films to improve brightness uniformity. Three different luminous intensity units, 120° mini-CSPLED, 150° mini-CSPLED, and 180° mini-CSPLED with different emission angle structures were fabricated using a CSP process. In terms of component characteristics, although the 180° mini-CSPLED light output power is about loss 4% (at 10 mA) compared with 150° mini-CSPLED, it has a large emission angle that forms a planar light source that contributes to improving the BLU brightness uniformity and reduced quantity of LEDs at the same area. In terms of BLU analysis, the blue mini-CSPLEDs with different emission angles excite the different QD film thicknesses; the chromaticity coordinates conversion to the white light region. The BLU brightness increases as the QD film thickness increases from 60, 90, and 150 μm. This result can achieve a brightness uniformity of 86% in a 180° mini-CSPLED BLU + 150-μm-thick QD films as compared to the 120° mini-CSPLED BLU and 150° mini-CSPLED BLU.

**Electronic supplementary material:**

The online version of this article (10.1186/s11671-019-2993-z) contains supplementary material, which is available to authorized users.

## Background

Liquid crystal display (LCD) is gaining greater favor as the mainstream display technology in various contemporary society fields. With the improvement of living standards, people have higher and higher requirements on LCD display quality. Especially in terms of color gamut and brightness, LCDs are constantly being surpassed by other display technologies such as organic light-emitting diodes (OLEDs) and laser displays [1–3]. In order to improve LCD performance, light-emitting diodes (LEDs) have gradually replaced the traditional cold cathode fluorescent lamp (CCFL) due to its small size, low energy consumption, and low heat generation. LEDs have become the new generation of LCD backlight unit (BLU) source [4–6]. At present, the LED BLU uses a blue LED to excite the yellow phosphor to form a white backlight. However, the low efficiency of the phosphor, the wide spectrum, the large light decay, and the poor uniformity of the particles hinder the brightness improvement and CIE chromaticity range of the LCD; thus, there is still room for improvement. It is well known that white LEDs are mainly fabricated by coating a YAG yellow phosphor layer onto a gallium nitride (GaN) blue LED (wavelength 450–470 nm) [7, 8]. However, its emission spectrum lacks red light, emits cool white light, is not natural enough, and has poor color rendering (CRI less than 75), which limits its application in high-end lighting and special fields. In order to obtain high CRI LEDs, a small amount of red phosphor and a small amount of green phosphor are added to the yellow phosphor to compensate and change the spectrum [6, 9]. However, this method of LED coated with phosphor is still insufficient in terms of luminous efficiency and chemical stability, and it is difficult to obtain large-scale popularization and application.

As a new type of fluorescent semiconductor nanocrystals, nano quantum dots (QDs) have many unique optical properties, such as high photoluminescence quantum yield, narrow emission spectrum, tunable emission spectrum, and high color purity [10–16]. It has been demonstrated that in the efficient photon management, QD converter can be widely used in solar cells [17, 18], LEDs [19, 20], and photodetectors [21–23]. Especially, QD photodetectors with selectable wavelengths and high responsivity and on/off ratio have been reported [24, 25]. Recently, QDs were also applied for water splitting due to its superior electrocatalytic and photocatalytic properties [26]. QDs have become a suitable candidate material in the display field, which has great potential to replace the traditional phosphor powder and increase the LCD color gamut range [27, 28]. QD-based backlight technology is currently the mainstream application target in displays, which have a great deal of attention from both the scientific and industrial circles. QDs are generally composed of groups II–VI or III–V elements and have a crystal grain diameter of only about 2–10 nm [29, 30]. Due to the quantum confinement effect, the QD energy gap can change with the particle size. In the past few years, research on cadmium selenide (CdSe) and its core-shell QDs in display technology has been the most popular, mainly because its light emission wavelength falls within the visible range. The QD-LED device structure is similar to that of a polymer light-emitting diode (PLED), and its emission layer is spin-coated using a colloidal semiconductor QDs solution, thereby having the advantages of the simple preparation process, low cost, and flexibility for fabrication [31–33].

At present, the mainstream LED BLU light source arrangement can be roughly divided into two types: edge-lit and direct-lit. In general, the contrast and brightness uniformity provided by the direct-lit will be better than the edge-lit. Edge-lit brightness uniformity uses a light guide plate to distribute light across the entire screen. However, the light guide plate weight becomes too great for large size LCD-TV applications. In addition, it needs to have good optical quality, resulting in high cost. Direct-lit does not use a light guide; the LED array is evenly placed directly below the LCD panel, which provides outstanding performance in brightness uniformity and good optical efficiency [34–36]. The BLU brightness and uniformity has a great influence on the display module uniformity. Therefore, it is very important to improve the BLU brightness uniformity. However, in actual applications, the BLU illumination uniformity is difficult to maintain. The brightness non-uniformity will be significantly different when the module becomes thinner. In order to achieve a thin LED and good uniformity, it is more challenging to design a BLU that meets the requirement. This study proposes a method to improve the LED BLU brightness uniformity. The BLU brightness uniformity was discussed through the different LED emission angles and the different QD film thicknesses.

## Methods

**Fig. 1:**
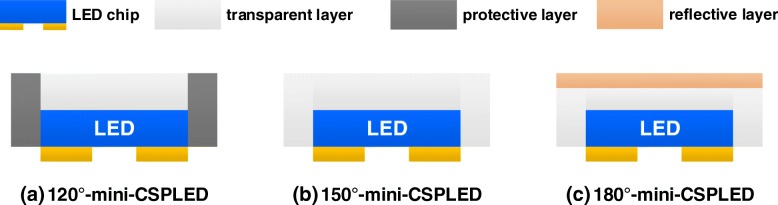
Schematic diagrams of three kinds of mini-CSPLED emission angle structure. **a** 120° mini-CSPLED, **b** 150° mini-CSPLED, and **c** 180° mini-CSPLED

**Fig. 2:**
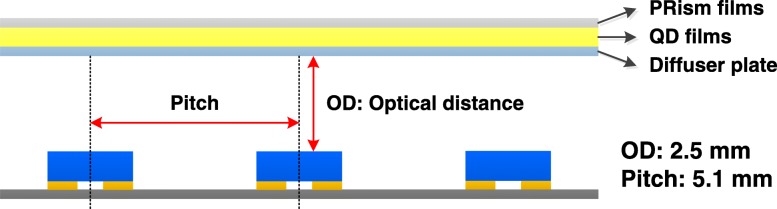
Schematic diagrams of the backlight unit structure

**Fig. 3:**
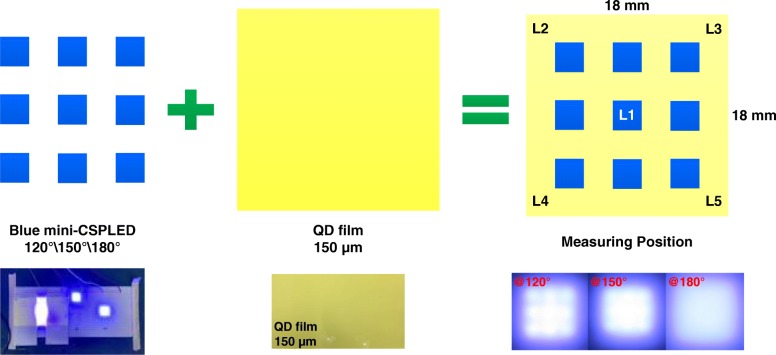
Schematic diagrams of the brightness uniformity measurement

The GaN LED epiwafer with an emission wavelength of 460 nm was grown by metal–organic chemical vapor deposition (MOCVD) on a c-plane sapphire substrate. The LED structure consists of a 2-μm-thick undoped GaN layer, a 2.0-μm-thick Si-doped n-type GaN cladding layer, six periods of InGaN/GaN multiple quantum wells (MQW), a 25-nm-thick Mg-doped p-AlGaN electron blocking layer, and a 0.2-μm-thick Mg-doped p-type GaN cladding layer. The Ni/Ag/Ni/Pt layers for ohmic contact layer and reflector were deposited onto the LED via electron beam evaporating system. Three different emission angle mini FC-LED (mini-LED) structures used in this study were fabricated by film transfer technique and molded chip scale package (CSP) method, with a detailed comparison: 120° mini-CSPLED, 150°mini-CSPLED, and 180°mini-CSPLED, as shown in Fig. 1. The 120° mini-CSPLED structure has a protective layer on all four sides of the chip and a transparent layer on the light emission surface. The 150° mini-CSPLED structure has a transparent layer on the side and the light emission surface of the chip. The 180° mini-CSPLED structure has a transparent layer on the side and the light emission on the chip surface, with a diffusion reflective layer covered onto the topmost layer. Where the material source of the transparent layer is the TiO_2_/silicone resin nanocomposite, both the thick protective layer and the thin diffusion reflective layer are the TiO_2_ powders. QD films were fabricated using CdSe/ZnS core-shell QDs as the material source. The green-emission (~ 525 nm) and red-emission (~ 617 nm) CdSe/ZnS core-shell QDs were mixed with polymethylmethacrylate (PMMA) to prepare various QD film thicknesses, in which the QD film optical characteristics can be found in Additional file 1: Figure S1. These QD films were fabricated as a color converter onto a LED chip (*λ* = 450 nm) to obtain white light devices. Figure 2 displays the BLU structure (18 mm × 18 mm), which consists of 3 × 3 square-shaped mini-LED array, diffusion plate, QD films, and two prism films. The mini-LED array was mounted onto a circuit board with a chip size of 20 mil ×20 mil and a pitch length of 5.1 mm. The effective optical distance (OD), by considering between chip and diffusion plate, is set as 2.5 mm in order to obtain good spatial uniformity. Figure 3 shows a blue mini LED array to excite QD films of different thicknesses (for instance, 60-μm-, 90-μm-, and 150-μm-thick QD films) to obtain a white planar light source. The brightness uniformity of the entire panel is evaluated as shown in Fig. 3 by measuring brightness in five points, L1–L5, located on the panel. The BLU brightness uniformity in this study is expressed by the following formula:

1 $$ \mathrm{Brightness}\ \mathrm{uniformity}=\frac{\mathrm{L}1+\mathrm{L}2+\mathrm{L}3+\mathrm{L}4+\mathrm{L}5}{5} $$

The light output power–current–voltage (*L–I–V*) characteristics of these mini-CSPLED was measured at room temperature using a Keithley 2400 source meter and an integrated sphere with a calibrated power meter (CAS 140B, Instrument Systems, Munich). The spatial radiation patterns of these mini-CSPLEDs were measured using a goniophotometer (LEDGON-100, Instrument Systems, Munich). The BLU luminance and electroluminescence (EL) spectra with QD films were analyzed using a spectral luminance meter (SRI-RL-5000, Optimum Optoelectronics Corp., Taiwan).

## Results and Discussion

**Fig. 4:**
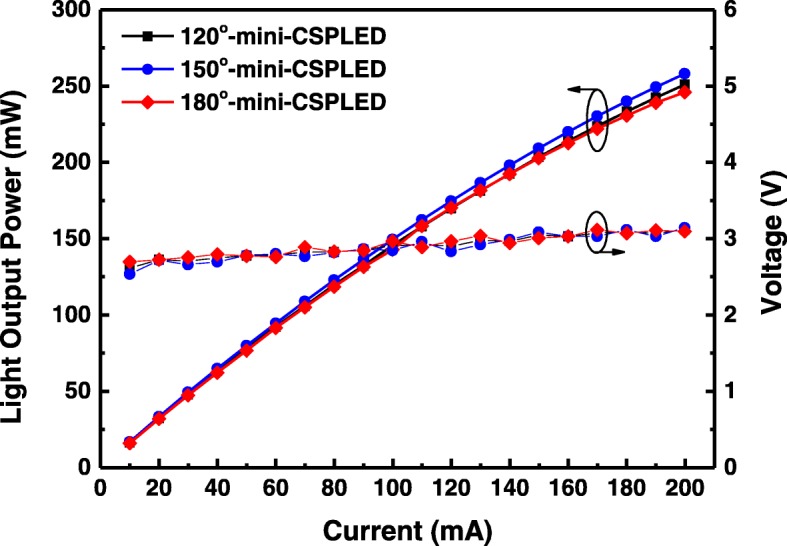
The *L–I–V* characteristics of the 120° mini-CSPLED, 150° mini-CSPLED, and 180° mini-CSPLED

Figure 4 presents the measured *L–I–V* characteristics for the three kinds of mini-CSPLED. At an injection current of 20 mA, the forward voltages of the 120° mini-CSPLED, 150° mini-CSPLED, and 180° mini-CSPLED were all the same and ∼ 2.72 V. Further increasing the injection current to 200 mA, the forward voltages of these three types mini-CSPLED were all increased to 3.09–3.14 V. It is clear that the *I–V* curves of these three devices are almost identical, demonstrating that the CSP process does not damage the electrical properties. On the other hand, the *L–I* curve shows only a slight difference in the light output power of the 120° mini-CSPLED, 150° mini-CSPLED, and 180° mini-CSPLED, which indicates the result of successful device optimization via the CSP structure. On the other hand, the light output power of the three kinds of mini-CSPLEDs initially increases linearly with the injection current. The *L–I* curve shows only a slight difference in the light output power of the 120° mini-CSPLED, 150° mini-CSPLED, and 180° mini-CSPLED, which indicates the result of successful device optimization via the CSP structure. As the injection current increased up to 200 mA, the light output power of the three kinds of mini-CSPLEDs were approximately 250.9, 258.0, and 245.9 mW. The light output power of the 120° mini-CSPLED exhibits lower than 150° mini-CSPLED, which may be absorbed by the diffusion reflective layer. The 180° mini-CSPLED gives 2.05% and 4.93% deterioration in the light output power at a high current of 200 mA as compared to the 120° mini-CSPLED and 150° mini-CSPLED. The deterioration could be attributed to the addition of a diffusion reflective layer on top of the transparent layer/CSPLED, the light may be slightly absorbed, or most of the light is concentrated in the transparent layer, with the reflection emitted from the sidewall.

**Fig. 5:**
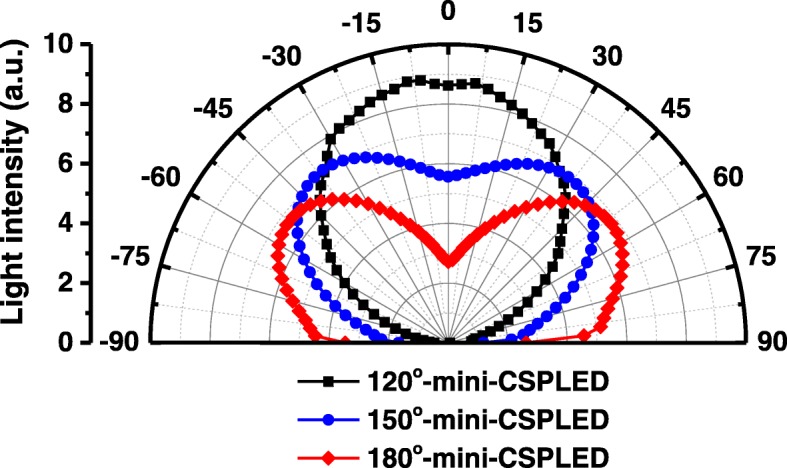
Radiation patterns of the 120° mini-CSPLED, 150° mini-CSPLED, and 180° mini-CSPLED (at 10 mA)

Figure 5 shows the radiation patterns of the 120° mini-CSPLED, 150° mini-CSPLED, and 180° mini-CSPLED at an injection current of 100 mA. The radiation pattern of mini-CSPLEDs can be controlled by varying the package structures. The 120° mini-CSPLED, 150° mini-CSPLED, and 180° mini-CSPLED viewing angles were measured to be 110.6°, 148.7°, and 180°, respectively. Obviously, the viewing angle of the 180° mini-CSPLED radiation pattern was larger than that of the 120° mini-CSPLED and 150° mini-CSPLED. It can be found that the central light output intensity of the radiation pattern of the 180° mini-CSPLED was depressed to half due to the diffusion reflective layer on the top. The wider viewing angle was caused by much light escape from the transparent layer after being reflected by the diffusion reflective layer, i.e., emission pattern with a butterfly wing-shaped light distribution; thus, it can be used as a planar light source. On the other hand, the 120° mini-CSPLED was covered with a diffusion reflective layer on all four sides, so that the light was concentrated and emitted upwards to form a Lambertian shaped light distribution. In addition, due to the five-sided conformal covered with a transparent layer, the light distribution of the 150° mini-CSPLED was similar to the batwing-shaped light distribution.

Table 1 shows the optoelectronic properties of the mini-CSPLED blue BLUs with different emission angles. With the same forward voltage of 24 V (at 10 mA), the CIE chromaticity coordinates (*x*, *y*) of the 120° mini-CSPLED BLU, the 150° mini-CSPLED BLU, and the 180° mini-CSPLED BLU were all similar and (*x*, *y*) = (*x* = 0.1518 − 0.15.2, *y* = 0.026 − 0.0281). Furthermore, the light output powers of the 120° mini-CSPLED blue BLU, 150° mini-CSPLED blue BLU, and 180° mini-CSPLED blue BLU were measured at 147.43, 153.02, and 146.71 mW, respectively. Due to the 180° mini-CSPLED package structure factor, the light output power was slightly poor, but the illumination area was increased.

**Table 1:** Optoelectronic properties of the three kinds of mini LED blue BLU

	120^o^	150^o^	180^o^
Chip size	20 mil × 20 mil		
Package size	1 mm × 1 mm		
Vf (*V*)	24	24	24
If (mA)	10	10	10
*x*	0.1518	0.1507	0.1502
*y*	0.026	0.0276	0.0281
Light output power (mW)	147.43	153.02	146.71

**Fig. 6:**
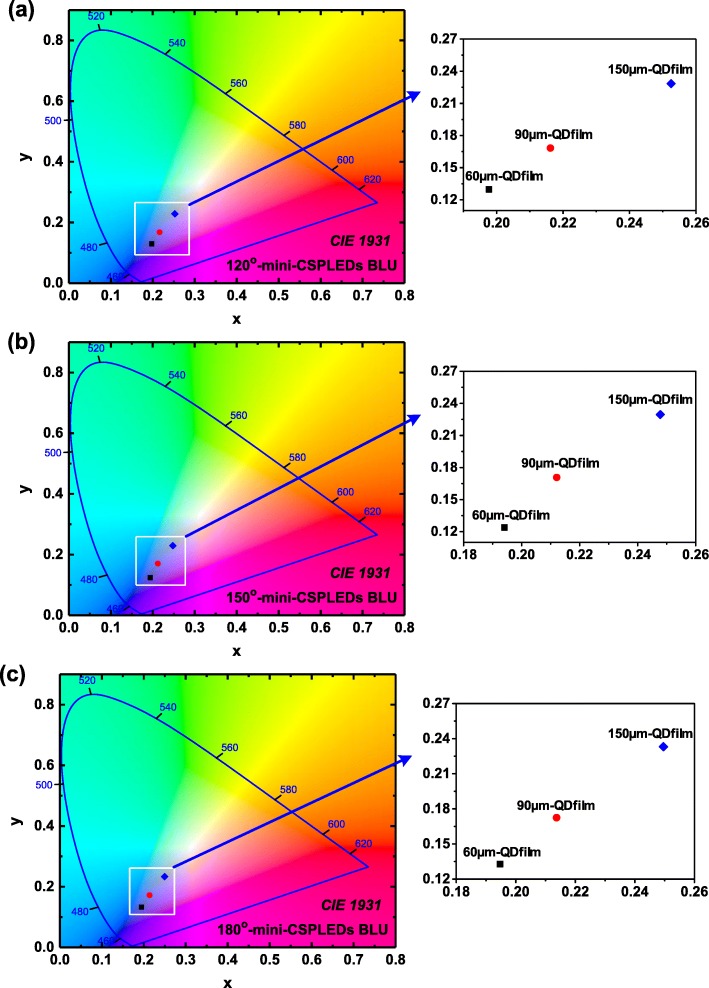
CIE chromaticity diagrams of 120° mini-CSPLED BLU, 150° mini-CSPLED BLU, and 180° mini-CSPLED BLU with different QD film thickness

Figures 6a–c show the CIE chromaticity diagram of 120° mini-CSPLED BLU, 150° mini-CSPLED BLU, and 180° mini-CSPLED BLU with different QD film thicknesses. The CIE chromaticity coordinates (*x*, *y*) of the three kinds of mini-CSPLED BLUs with different QD film thicknesses were measured as follows:(*x*, *y*) = (*x* = 0.1977 − 0.2525, *y* = 0.1297 − 0.2284), (*x*, *y*) = (*x* = 0.1941 − 0.2478, *y* = 0.1239 − 0.2295), and (*x*, *y*) = (*x* = 0.1947 − 0.2496, *y* = 0.1328 − 0.2331), respectively. It was clear that the emission chromaticity coordinates of the corresponding BLU with QD films of various thicknesses exhibiting CIE chromaticity coordinates were located near the blue region. As the QD film thickness increases, the CIE chromaticity coordinates shift toward the white region. In addition, the BLU brightness increases as the QD film thickness increases from 60, 90, and 150 μm. This result was attributed to the significant increases the excitation probability with thick QD films to produce white light and increase brightness. On the other hand, the BLU brightness of the 180° mini-CSPLED BLU was significantly lowered, which may be attributed to the average brightness decrease as a result of the larger illumination area. The results of this study show the CIE chromaticity coordinates (*x*, *y*) and brightness for the three kinds of mini-CSPLED emission angle structure with different QD film thicknesses and are summarized in Tables 2, 3, and 4, in which the data measurement can be found in Additional file 1: Figures S2–S10.

**Table 2:** The CIE chromaticity coordinates (*x*, *y*) and brightness for the 120°mini-CSPLEDs with different QD film thicknesses

120° mini-CSPLED			
QD film thickness (μm)	60	90	150
*x*	0.1977	0.2162	0.2525
*y*	0.1297	0.1683	0.2284
Brightness (cd/m^2^)	8,532	9,561	9,900

**Table 3:** The CIE chromaticity coordinates (*x*, *y*) and brightness for the 150° mini-CSPLEDs with different QD film thicknesses

150° mini-CSPLED			
QD film thickness (μm)	60	90	150
*x*	0.1941	0.2121	0.2478
*y*	0.1239	0.1707	0.2295
Brightness (cd/m^2^)	9,638	11,331	10,319

**Table 4:** The CIE chromaticity coordinates (*x*, *y*) and brightness for the 180° mini-CSPLEDs with different QD film thicknesses

180° mini-CSPLED			
QD film thickness (μm)	60	90	150
*x*	0.1947	0.2137	0.2496
*y*	0.1328	0.1725	0.2331
Brightness (cd/m^2^)	7,365	8,463	8,645

**Fig. 7:**
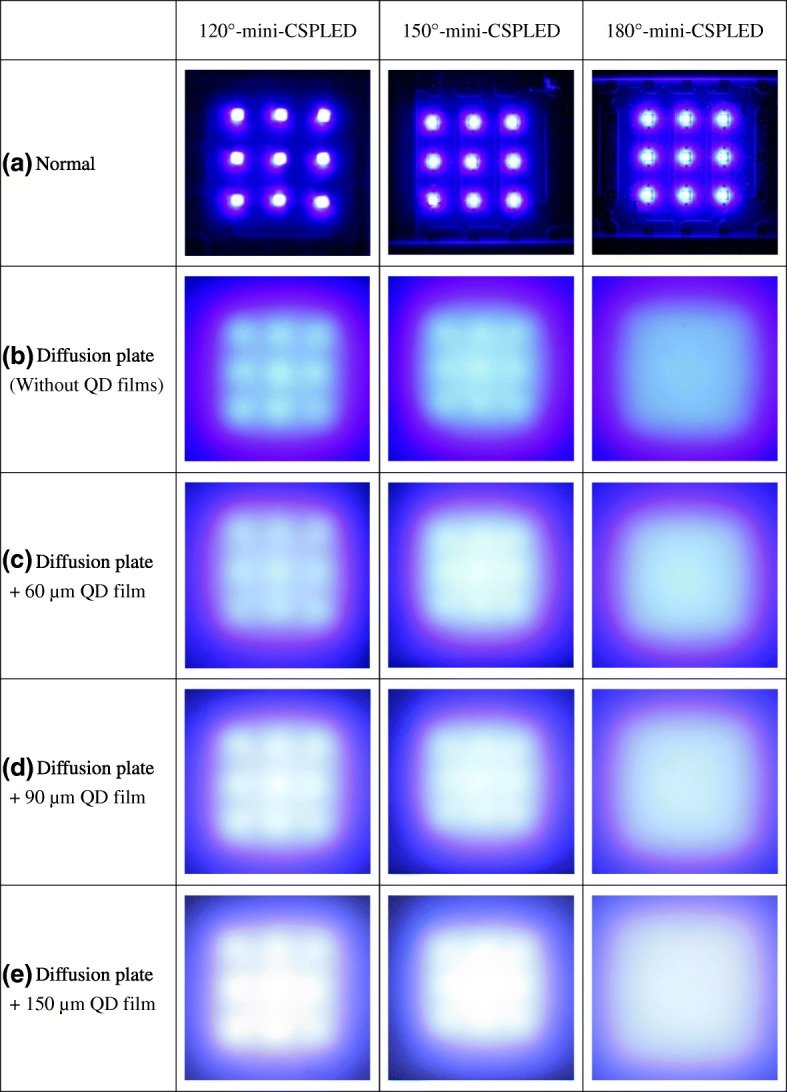
Light distribution images of 120° mini-CSPLED BLU, 150° mini-CSPLED BLU, and 180° mini-CSPLED BLU with and without a diffusion plate and different QD film thickness

Figures 7a–e show the light distribution images of 120° mini-CSPLED BLU, 150° mini-CSPLED BLU, and 180° mini-CSPLED BLU with and without a diffusor and different QD film thicknesses. Figure 7a shows the light distribution images of the three kinds of mini-CSPLED blue BLUs without diffusor and QD films. By placing the diffusion plate on the three kinds of mini-CSPLED BLUs, it can be seen that the 180° mini-CSPLED BLU has a better uniform planar light compared to the 120° mini-CSPLED BLU and 150° mini-CSPLED BLU. However, the 120° mini-CSPLED BLU and the 150° mini-CSPLED BLU show the stripe patterns, in which the 120° mini-CSPLED BLU is the most visible, as shown in Fig. 4b. Similarly, as shown in Figs. 7c–e, the QD films are placed on the diffusion plate, and as the QD film thickness is increased, the light distribution images of the three kinds of mini-CSPLED BLUs clearly presented that the BLU brightness is increased and is closer to white light; the stripe pattern is also less and less unobvious. The light distribution images observations are in good agreement with the CIE chromaticity coordinates (*x*, *y*) and brightness results.

From the above results, it can be seen that the CIE color coordinates (*x*, *y*) can be brought close to the white region using 150-μm-thick QD films. Therefore, the thickness of QD films was fixed, and the effects of brightness uniformity of the three kinds of mini-CSPLED BLUs were discussed. Using the 5-point brightness uniformity measurement method, the brightness uniformity of the three kinds of mini-CSPLED BLU + 150-μm-thick QD films were estimated to be 35%, 39%, and 86%, respectively. Obviously, there were 1.47 times and 1.19 times improvement in the BLU brightness uniformity of 180° mini-CSPLED BLU compared with that of the 120° mini-CSPLED BLU and 150° mini-CSPLED BLU. Therefore, it was found that the use of 180° mini-CSPLED BLU +150-μm-thick QD film could effectively improve the overall BLU brightness uniformity. The brightness uniformity calculation of the three kinds of mini-CSPLED BLUs + 150-μm-thick QD films was summarized in Table 5, in which the data measurement can be found in Additional file 1: Figures S11–S22.

**Table 5:** Brightness uniformities of three kinds of mini-CSPLED BLUs with 150-μm-thick QD films

Mini-CSPLED type	120°	150°	180°
L1 (center) (cd/m^2^)	9,900	10,319	8,645
L2 (cd/m^2^)	1,803	2,557	7,110
L3 (cd/m^2^)	1,393	2,385	7,227
L4 (cd/m^2^)	2,368	2,405	7,226
L5 (cd/m^2^)	1,783	2,652	7,050
L1 (center) (%)	100%	100%	100%
L2 (%)	18%	25%	82%
L3 (%)	14%	23%	84%
L4 (%)	24%	23%	84%
L5 (%)	18%	26%	82%
Brightness uniformity (%)	35%	39%	86%

**Fig. 8:**
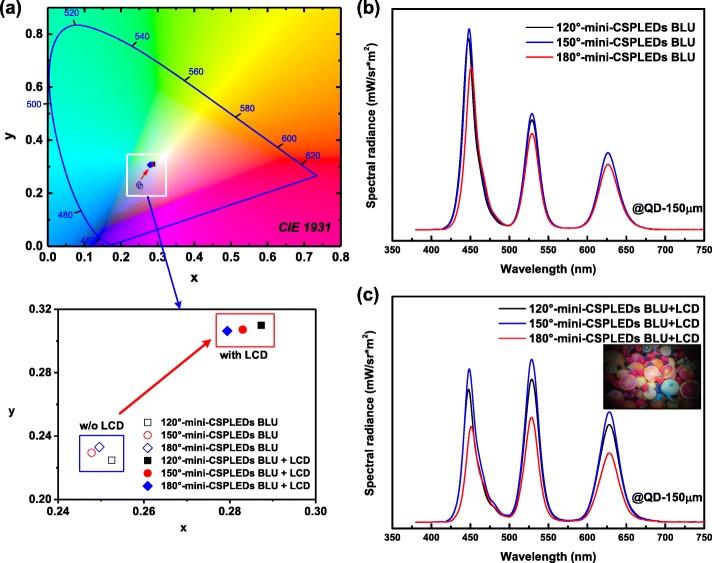
**a** CIE chromaticity diagram. **b**, **c** EL spectra of three kinds of mini-CSPLED BLUs + 150 μm-thick QD films with and without LCD

Figures 8a–c show the CIE chromaticity diagram and EL spectra of the three kinds of mini-CSPLED BLUs + 150 μm-thick QD films with and without LCD. As shown in Fig. 8a, it can be seen that the CIE chromaticity coordinate (*x*, *y*) of the 120° mini-CSPLED BLU with LCD shifted from (0.2525, 0.2284) to (0.2873, 0.3099). The 150° mini-CSPLED BLU with LCD was from (0.2478, 0.2295) to (0.2830, 0.3072). The 180° mini-CSPLED BLU with LCD was from (0.2496, 0.2331) to (0.2794, 0.3063). This shows that with the addition of LCD, the CIE chromaticity coordinates shifted more toward the white region. The EL spectrum of the three kinds of mini-CSPLED BLUs + 150-μm-thick QD films without LCD exhibits a strong blue light intensity, and the CIE chromaticity coordinate is located in the near blue region, as shown in Fig. 8b (see Additional file 1: Figures S4, S7, and S10). When the LCD was placed on the three kinds of mini-CSPLED BLUs + 150 μm-thick QD films, the EL spectrum shows that the red, green, and blue light intensity were similar, and the CIE chromaticity coordinate was located in the white region. This result can be attributed to the color filter of the LCD structure, which improves the color coordinate position, as shown in Fig. 8c (see Additional file 1: Figures S23–S25). The inset shows the actual application photograph of 180° mini-CSPLED BLU + 150-μm-thick QD film with LCD.

## Conclusions

In conclusion, we successfully used mini-CSPLED BLU as the blue light and excitation energy source, along with QD films, to produce a uniform white backlight. Mini-CSPLEDs were subjected to emission angle packaged structure fabricate for 120°, 150°, and 180° to verify that the mini-CSPLED optical performance had significant differences. The lager emission angle and an illumination area of 180° mini-CSPLED improved significantly when compared with that of the 120° mini-CSPLED and 150° mini-CSPLED. Impressively, 180° mini-CSPLED BLU with a 150-μm-thick QD film achieved an excellent uniform brightness planar white light source for backlight displays of approximately 86%, which is significant for the future ultra-thin display technology. We implemented highly reliable CSP technology that can protect the LED chip, solve the emission angle and illumination area problems of LEDs, and fabricate a backlight source for displays with good brightness uniformity.

## Additional File

Additional file 1: **Figure S1.** QD films characteristics – PL spectrum (excited by a 405 nm laser). **Figure S2.** Spectroradiometric report of 120°-mini-CSPLED with a 60 μm-thick QD films. **Figure S3.** Spectroradiometric report of 120°-mini-CSPLED with a 90 μm-thick QD films. **Figure S4.** Spectroradiometric report of 120°-mini-CSPLED with a 150 μm-thick QD films. **Figure S5.** Spectroradiometric report of 150°-mini-CSPLED with a 60 μm-thick QD films. **Figure S6.** Spectroradiometric report of 150°-mini-CSPLED with a 90 μm-thick QD films. **Figure S7.** Spectroradiometric report of 150°-mini-CSPLED with a 150 μm-thick QD films. **Figure S8.** Spectroradiometric report of 180°-mini-CSPLED with a 60 μm-thick QD films. **Figure S9.** Spectroradiometric report of 180°-mini-CSPLED with a 90 μm-thick QD films. **Figure S10.** Spectroradiometric report of 180°-mini-CSPLED with a 150 μm-thick QD films. **Figure S11.** The L2 spectroradiometric report of 120°-mini-CSPLED with a 150 μm-thick QD films. **Figure S12.** The L3 spectroradiometric report of 120°-mini-CSPLED with a 150 μm-thick QD films. **Figure S13.** The L4 spectroradiometric report of 120°-mini-CSPLED with a 150 μm-thick QD films. **Figure S14.** The L5 spectroradiometric report of 120°-mini-CSPLED with a 150 μm-thick QD films. **Figure S15.** The L2 spectroradiometric report of 150°-mini-CSPLED with a 150 μm-thick QD films. **Figure S16.** The L3 spectroradiometric report of 150°-mini-CSPLED with a 150 μm-thick QD films. **Figure S17.** The L4 spectroradiometric report of 150°-mini-CSPLED with a 150 μm-thick QD films. **Figure S18.** The L5 spectroradiometric report of 150°-mini-CSPLED with a 150 μm-thick QD films. **Figure S19.** The L2 spectroradiometric report of 180°-mini-CSPLED with a 150 μm-thick QD films. **Figure S20.** The L3 spectroradiometric report of 180°-mini-CSPLED with a 150 μm-thick QD films. **Figure S21.** The L4 spectroradiometric report of 180°-mini-CSPLED with a 150 μm-thick QD films. **Figure S22.** The L5 spectroradiometric report of 150°-mini-CSPLED with a 150 μm-thick QD films. **Figure S23.** Spectroradiometric report of 120°-mini-CSPLED + 150 μm-thick QD films with LCD. **Figure S24.** Spectroradiometric report of 150°-mini-CSPLED + 150 μm-thick QD films with LCD. **Figure S25.** Spectroradiometric report of 180°-mini-CSPLED + 150 μm-thick QD films with LCD. (DOCX 3461 kb)

## Abbreviations

BLU: Backlight unit
CCFL: Cold cathode fluorescent lamp
CdSe: Cadmium selenide
GaN: Gallium nitride
LCD: Liquid crystal display
mini-CSPLED: Mini chip-scale packaged light-emitting-diode
OLED: Organic light-emitting diode
PLED: Polymer light-emitting diode
QDs: Quantum dots
YAG: Yttrium aluminum garnet

## References

[1] Chen HW, Tan GJ, Wu ST (2017). Ambient contrast ratio of LCDs and OLED displays. Opt Express.

[2] Tang CW, Vanslyke SA (1987). Organic electroluminescent diodes. Appl Phys Lett.

[3] Song H, Li H, Liu X (2018). Studies on different primaries for a nearly-ultimate gamut in a laser display. Opt Express.

[4] Lv X, Loo KH, Lai YM, Tse CK (2014). Energy-saving driver design for full-color large-area LED display panel systems. IEEE Trans Ind Electron..

[5] Templier F (2016). GaN-based emissive microdisplays: A very promising technology for compact, ultra-high brightness display systems. J Soc Inf Display.

[6] Wang L, Wang X, Kohsei T, Yoshimura K, Izumi M, Hirosaki N, Xie RJ (2015). Highly efficient narrow-band green and red phosphors enabling wider color-gamut LED backlight for more brilliant displays. Opt Express.

[7] Lin HT, Tien CH, Hsu CP, Horng RH (2014). White thin-film flip-chip LEDs with uniform color temperature using laser lift-off and conformal phosphor coating technologies. Opt Express.

[8] Tien CH, Ho KW, Chien HY, Wuu DS, Horng RH (2016). Effect of the phosphor permanent substrate on the angular CCT for white thin-film flip-chip light-emitting diodes. IEEE Trans Electron Devices.

[9] Yang SH, Lin JS, Juang FS, Chou DC, Chung MH, Chen CM, Liu LC (2013). White light emitting diodes (LEDs) with good color rendering indices (CRI) and high luminous efficiencies by the encapsulation of mixed and double-deck phosphors. Curr Appl Phys.

[10] Coe S, Woo W-K, Bawendi M, Bulović V (2002). Electrolumi-nescence from single monolayers of nanocrystals in molecularorganic devices. Nature.

[11] Colvin VL, Schlamp MC, Alivisatos AP (1994). Light-emitting diodes made from cadmium selenide nanocrystals and a semiconducting polymer. Nature.

[12] Chen HS, Hsu CK, Hong HY (2006). InGaN-CdSe-ZnSe quantum dots white LEDs. IEEE Photonics Technol Lett..

[13] Janssen RAJ, Stouwdam JW (2008). Red, green, and blue quantum dot LEDs with solution processable ZnO nanocrystal electron injection layers. J Mater Chem..

[14] Al-Amri AM, Cheng B, He J-H (2019). Perovskite methylammonium lead trihalide heterostructures: progress and challenges. IEEE Trans Nano Technol..

[15] Cheng B (2019). Orthogonal patterning halide perovskite for nanodevice application. ACS Nano.

[16] Tseng JY, Lee L, Huang YC, Chang JH, Su TY, Shih YC, Lin HW, Chueh YL (2018). Pressure welding of silver nanowires networks at room temperature as transparent electrodes for efficient organic light-emitting diodes. Small.

[17] Das S, Hossain MJ, Leung SF, Lenox A, Jung Y, Davis K, He JH, Roy T (2019). A leaf-inspired photon management scheme using optically tuned bilayer nanoparticles for ultra-thin and highly efficient photovoltaic devices. Nano Energy.

[18] Wang HP, Periyanagounder D, Li AC, He JH (2019). Fabrication of Silicon Hierarchical Structures for Solar Cell Applications. IEEE Access.

[19] Chen GH, Yeh CW, Yeh MH, Ho SJ, Chen HS (2015). Wide gamut white light emitting diodes using quantum dot-silicone film protected by an atomic layer deposited TiO_2_ barrier. Chem Commun..

[20] Choi MK, Yang J, Hyeon T, Kim DH (2018). Flexible quantum dot light-emitting diodes for next-generation displays. NPJ Flexible Electron..

[21] Malinowski PE, Georgitzikis E, Maes J, Vamvaka I, Frazzica F, Olmen JV, Moor PD, Heremans P, Hens Z, Cheyns D (2017). Thin-film quantum dot photodiode for monolithic infrared image sensors. Sensors.

[22] Yang W, Chen J, Zhang Y, Zhang Y, He JH, Fang X (2019) Silicon-compatible photodetectors: trends to monolithically integrate photosensors with chip technology. Adv Func Mater 1808182.

[23] Chen YZ, Wang SW, Su TY, Lee SH, Chen CW, Yang CH, Wang K, Kuo HC, Chueh YL (2018). Phase-engineered type-II multimetal–selenide heterostructures toward low-power consumption, flexible, transparent, and wide-spectrum photoresponse photodetectors. Small.

[24] Alarawi A, Ramalingam V, He JH (2019). Recent advances in emerging single atom confined two-dimensional materials for water splitting applications. Mater Today Energy.

[25] Tang SY, Medina H, Yen YT, Chen CW, Yang TY, Wei KH, Chueh YL (2019). Enhanced photocarrier generation with selectable wavelengths by M-decorated-CuInS2 nanocrystals (M = Au and Pt) synthesized in a single surfactant process on MoS2 bilayers. Small.

[26] Liang X, Dong R, Ho JC (2019). Self-assembly of colloidal spheres toward fabrication of hierarchical and periodic nanostructures for technological applications. Adv Mate Tech.

[27] Kim HJ, Shin MH, Kim YJ (2016). Optical efficiency enhancement in white organic light-emitting diode display with high color gamut using patterned quantum dot film and long pass filter. Jpn. J Appl Phys..

[28] Chung W, Park K, Yu HJ, Kim J, Chun BH, Kim SH (2010). White emission using mixtures of CdSe quantum dots and PMMA as a phosphor. Opt Mater.

[29] Xie R, Kolb U, Li J, Basché T, Mews A (2005). Synthesis and Characterization of Highly Luminescent CdSe-Core CdS/Zn0.5Cd0.5S/ZnS Multishell Nanocrystals. J Am Chem Soc.

[30] Baek J, Shen Y, Lignos I, Bawendi MG, Jensen KF (2018). Multistage microfluidic platform for the continuous synthesis of III–V core/shell quantum dots. Angew Chem Int Edit.

[31] Chung DY, Huang J, Bradley DDC, Campbell AJ (2010). High performance, flexible polymer light-emitting diodes (PLEDs) with gravure contact printed hole injection and light emitting layers. Org Electron.

[32] Zheng H, Zheng Y, Liu N, Ai N, Wang Q, Wu S, Zhou J, Hu D, Yu S, Han S, Xu W, Luo C, Meng Y, Jiang Z, Chen Y, Li D, Huang F, Wang J, Peng J, Cao Y (2013). All-solution processed polymer light-emitting diode display. Nat Commun.

[33] Niu Q, Shao Y, Xu W, Wang L, Han S, Liu N, Peng J, Cao Y, Wang J (2008). Full color and monochrome passive-matrix polymer light-emitting diodes flat panel displays made with solution processes. Org. Electron..

[34] West RS, Konijn H, Sillevis-Smitt W, Kuppens S, Pfeffer N, Martynov Y, Takaaki Y, Eberle S, Harbers G, Tan TW, Chan CE (2003). High brightness direct LED backlight for LCD-TV. SID Symposium Digest Tech..

[35] Sun CC, Moreno I, Chung SH, Chien WT, Hsieh CT, Yang TH (2008). Brightness management in a direct LED backlight for LCD TVs. J Soc Inf Disp..

[36] Yan JR, Li DC, Wang ZK, Rao WP (2016). An iterative method for the uniformity improvement of edge-lit backlight. Adv Opto Electron..

