# Supplementary material for: High-Uniformity Planar Mini-Chip-Scale Packaged LEDs with Quantum Dot Converter for White Light Source
Source: Nanoscale Res Lett. 2019 May 29;14:182. doi: 10.1186/s11671-019-2993-z (PMC6541665; doi:10.1186/s11671-019-2993-z)
Supplement: Supplementary file 1 — Figure S1. QD films characteristics – PL spectrum (excited by a 405 nm laser). Figure S2. Spectroradiometric report of 120°-mini-CSPLED with a 60 μm-thick QD films. Figure S3. Spectroradiometric report of 120°-mini-CSPLED with a 90 μm-thick QD films. Figure S4. Spectroradiometric report of 120°-mini-CSPLED with a 150 μm-thick QD films. Figure S5. Spectroradiometric report of 150°-mini-CSPLED with a 60 μm-thick QD films. Figure S6. Spectroradiometric report of 150°-mini-CSPLED with a 90 μm-thick QD films. Figure S7. Spectroradiometric report of 150°-mini-CSPLED with a 150 μm-thick QD films. Figure S8. Spectroradiometric report of 180°-mini-CSPLED with a 60 μm-thick QD films. Figure S9. Spectroradiometric report of 180°-mini-CSPLED with a 90 μm-thick QD films. Figure S10. Spectroradiometric report of 180°-mini-CSPLED with a 150 μm-thick QD films. Figure S11. The L2 spectroradiometric report of 120°-mini-CSPLED with a 150 μm-thick QD films. Figure S12. The L3 spectroradiometric report of 120°-mini-CSPLED with a 150 μm-thick QD films. Figure S13. The L4 spectroradiometric report of 120°-mini-CSPLED with a 150 μm-thick QD films. Figure S14. The L5 spectroradiometric report of 120°-mini-CSPLED with a 150 μm-thick QD films. Figure S15. The L2 spectroradiometric report of 150°-mini-CSPLED with a 150 μm-thick QD films. Figure S16. The L3 spectroradiometric report of 150°-mini-CSPLED with a 150 μm-thick QD films. Figure S17. The L4 spectroradiometric report of 150°-mini-CSPLED with a 150 μm-thick QD films. Figure S18. The L5 spectroradiometric report of 150°-mini-CSPLED with a 150 μm-thick QD films. Figure S19. The L2 spectroradiometric report of 180°-mini-CSPLED with a 150 μm-thick QD films. Figure S20. The L3 spectroradiometric report of 180°-mini-CSPLED with a 150 μm-thick QD films. Figure S21. The L4 spectroradiometric report of 180°-mini-CSPLED with a 150 μm-thick QD films. Figure S22. The L5 spectroradiometric report of 150°-mini-CSPLED with a 150 μm-thick QD films. Fig [file 11671_2019_2993_MOESM1_ESM.docx]

**Supplementary Information**

 Fig. S1. QD films characteristics – PL spectrum (excited by a 405 nm laser).


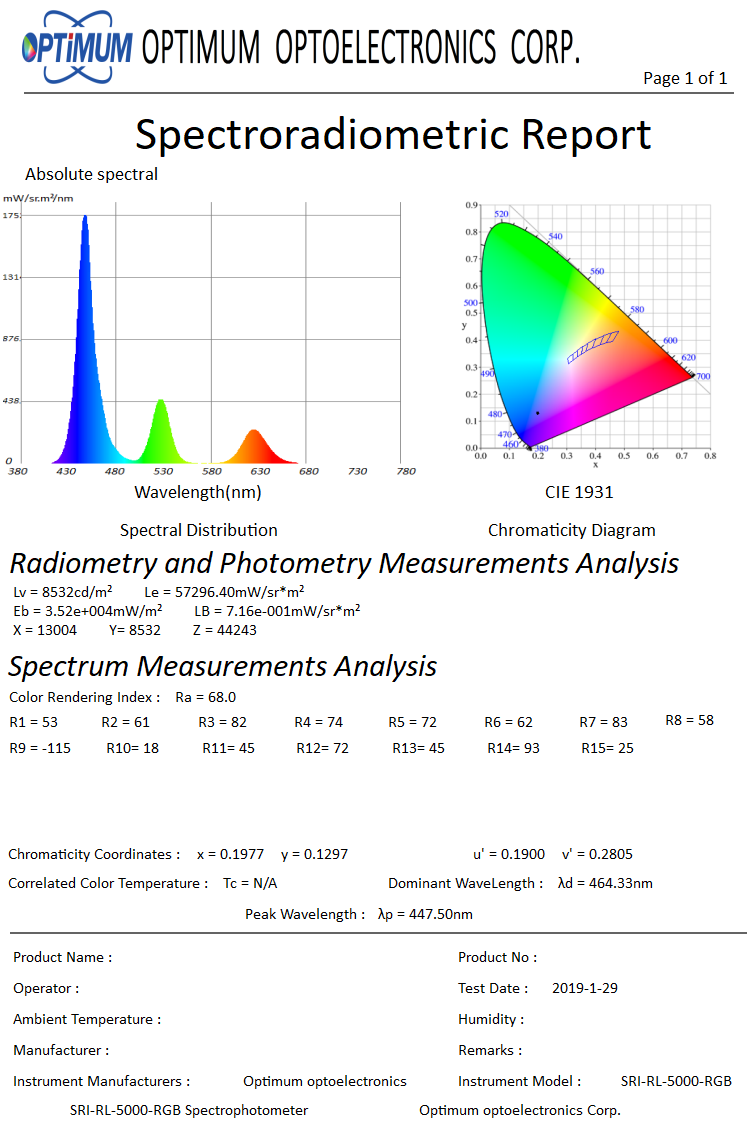


Fig. S2. Spectroradiometric report of 120°-mini-CSPLED with a 60 μm-thick QD films.


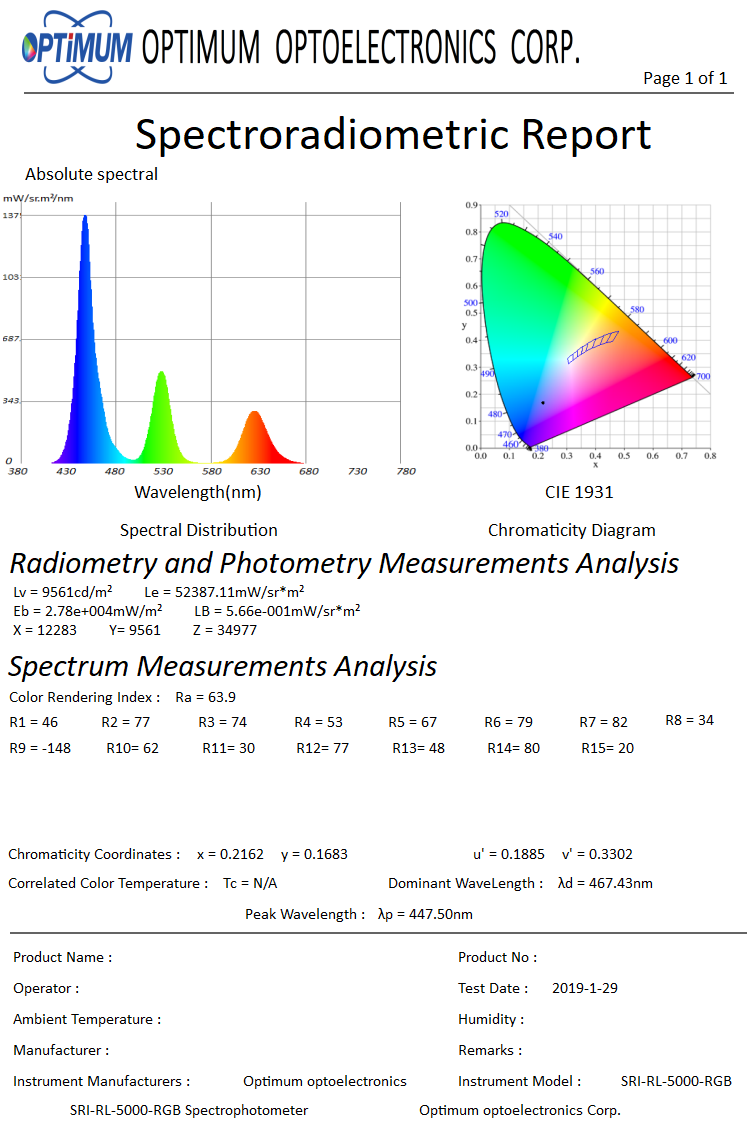


Fig. S3. Spectroradiometric report of 120°-mini-CSPLED with a 90 μm-thick QD films.


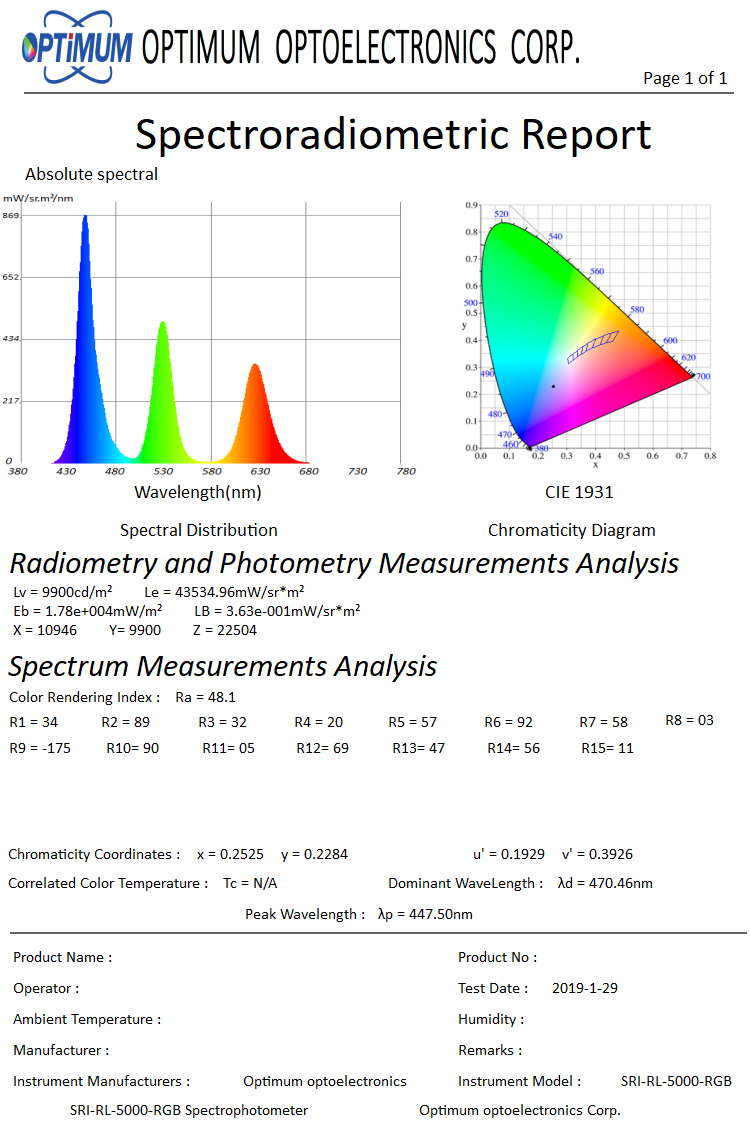


Fig. S4. Spectroradiometric report of 120°-mini-CSPLED with a 150 μm-thick QD films.


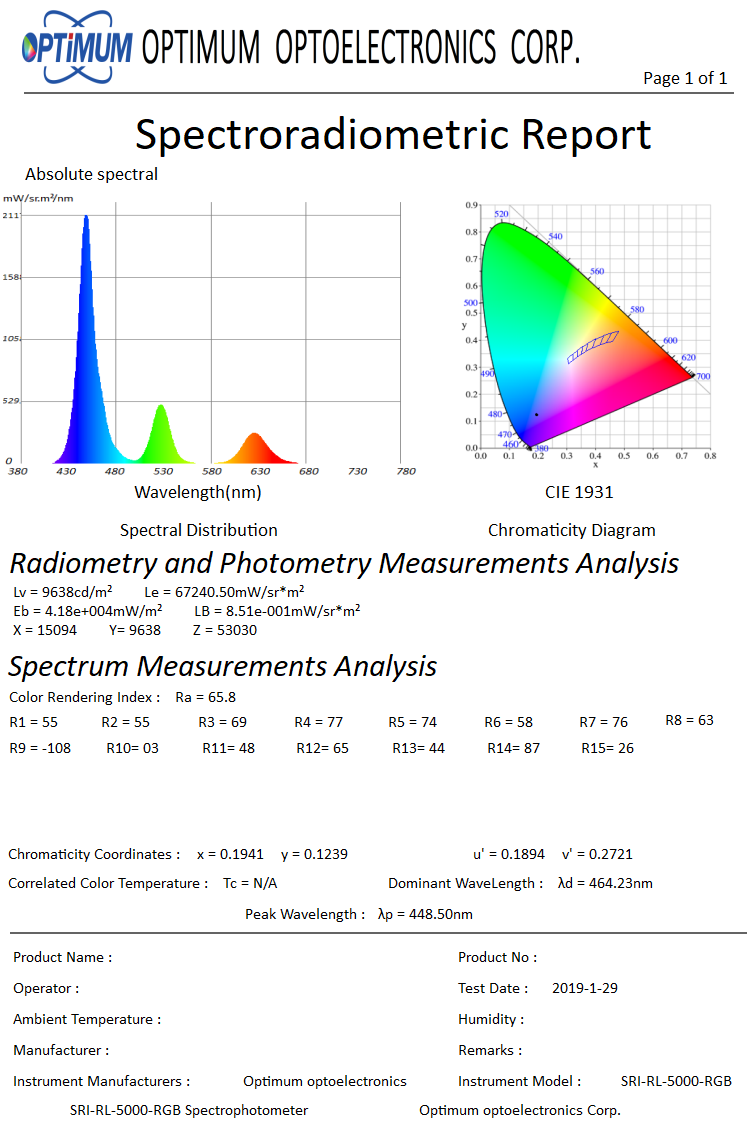


Fig. S5. Spectroradiometric report of 150°-mini-CSPLED with a 60 μm-thick QD films.


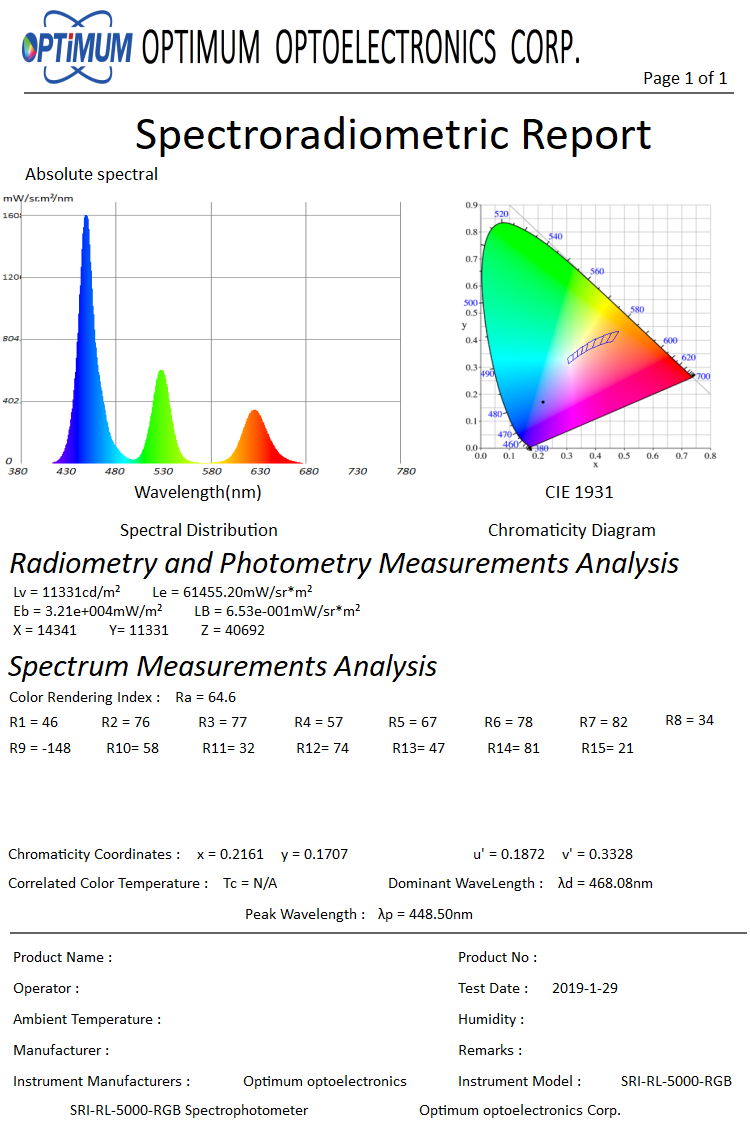


Fig. S6. Spectroradiometric report of 150°-mini-CSPLED with a 90 μm-thick QD films.


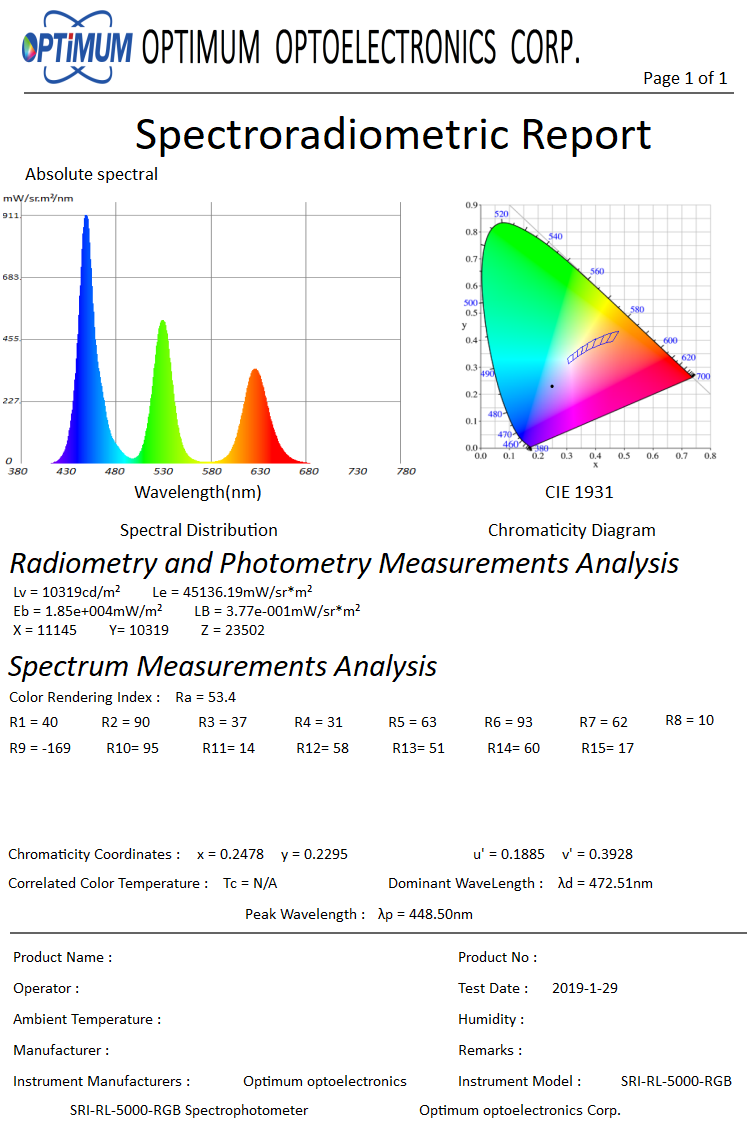


Fig. S7. Spectroradiometric report of 150°-mini-CSPLED with a 150 μm-thick QD films.


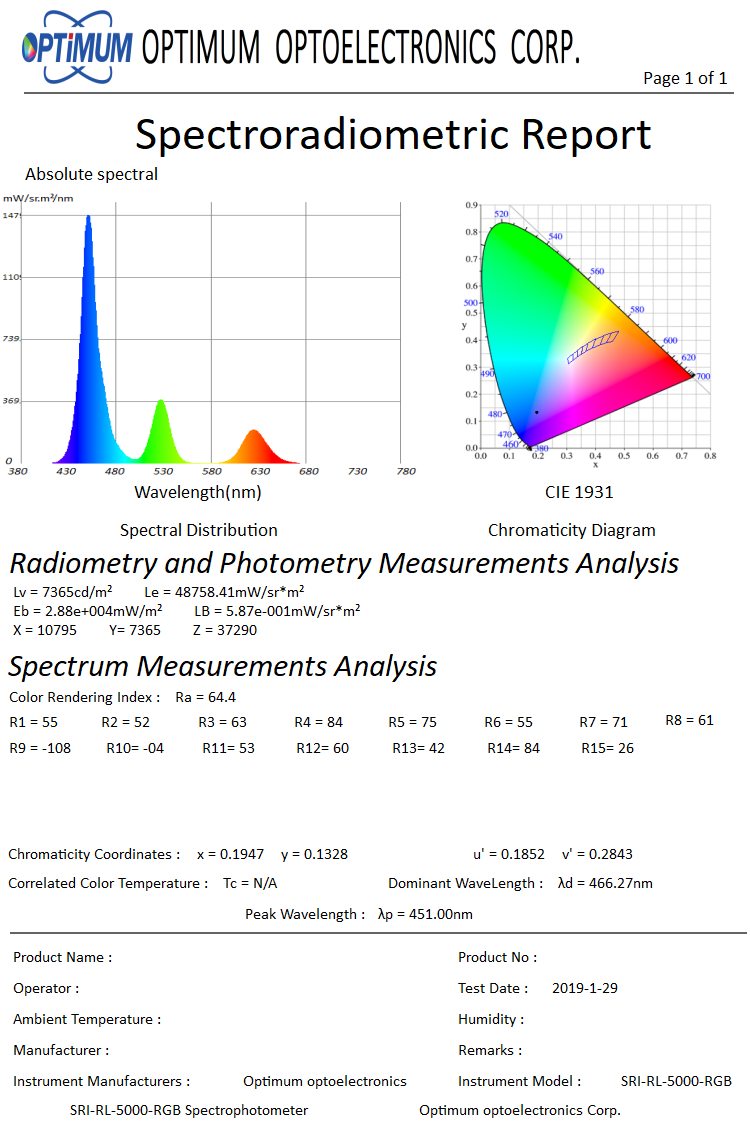


Fig. S8. Spectroradiometric report of 180°-mini-CSPLED with a 60 μm-thick QD films.


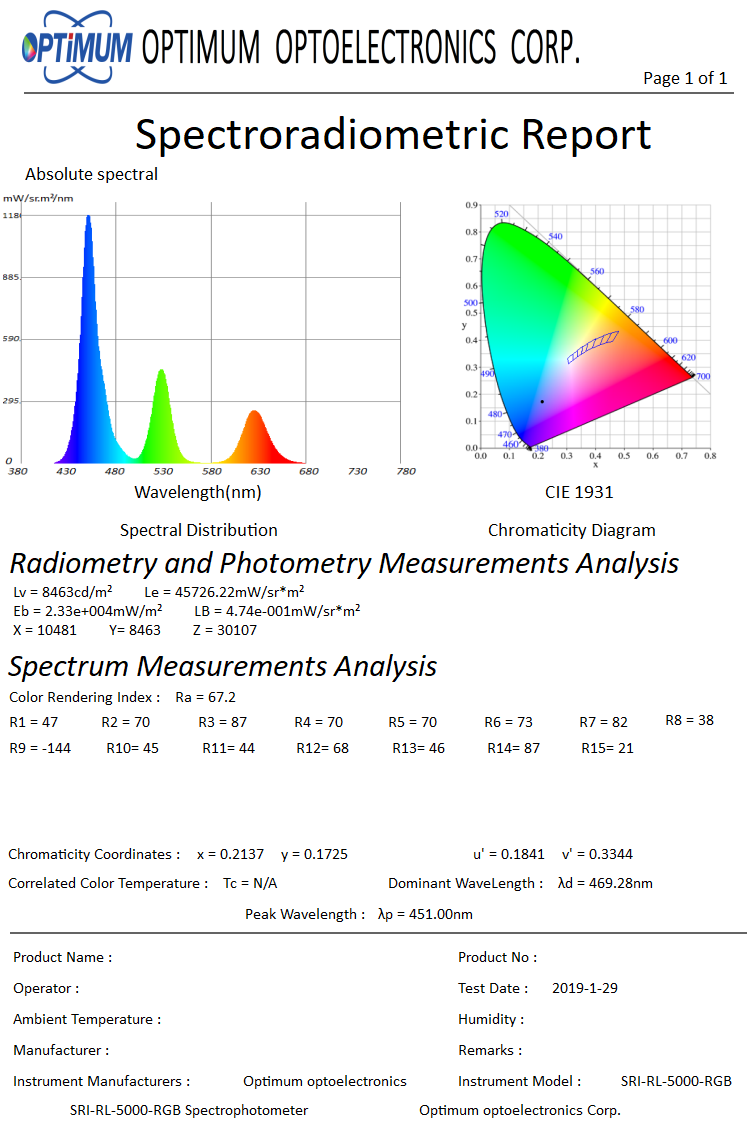


Fig. S9. Spectroradiometric report of 180°-mini-CSPLED with a 90 μm-thick QD films.


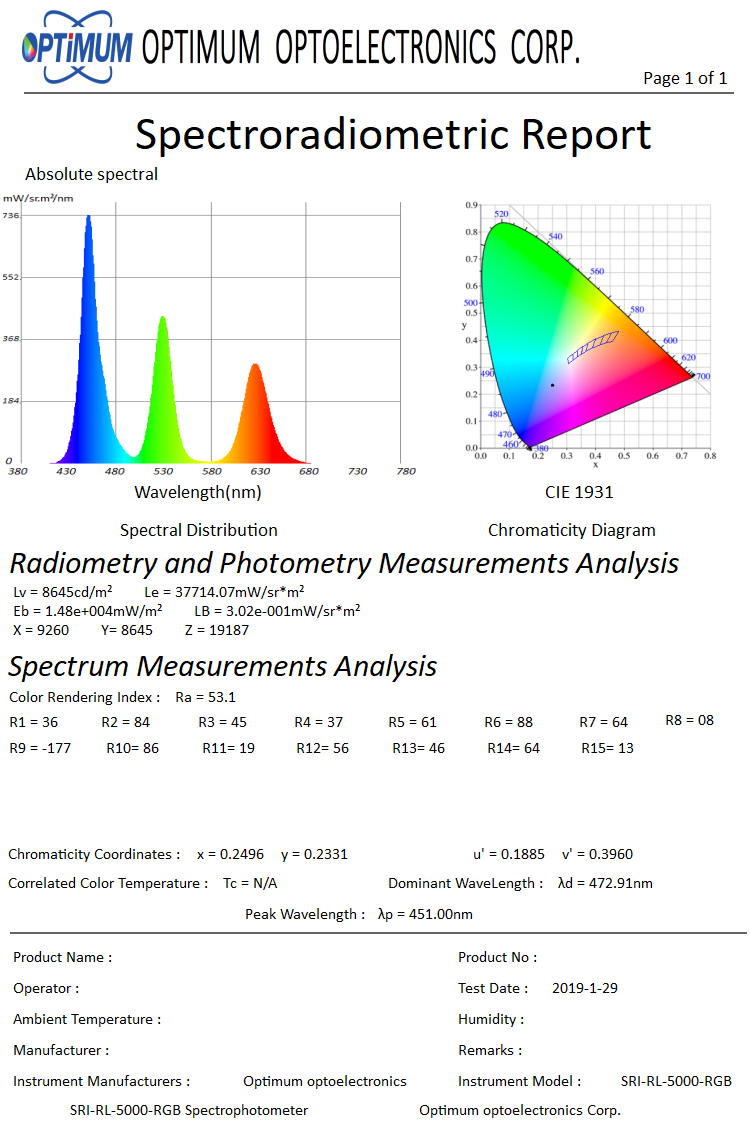


Fig. S10. Spectroradiometric report of 180°-mini-CSPLED with a 150 μm-thick QD films.


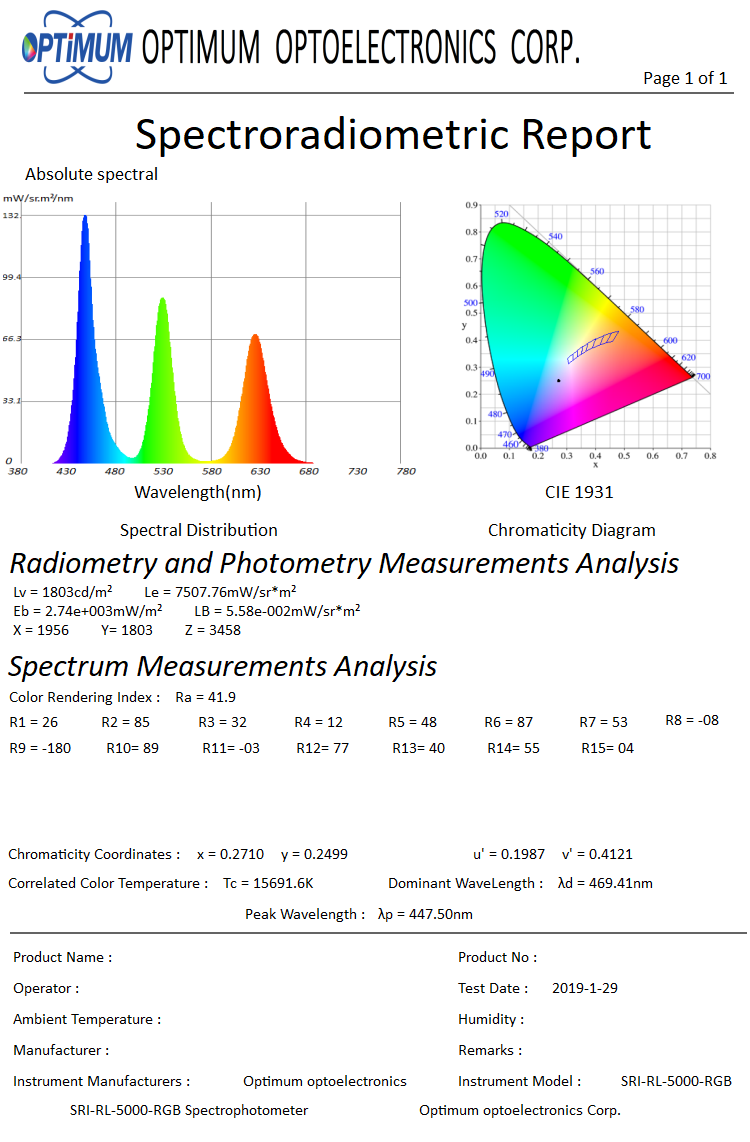


Fig. S11. The L2 spectroradiometric report of 120°-mini-CSPLED with a 150 μm-thick QD films.


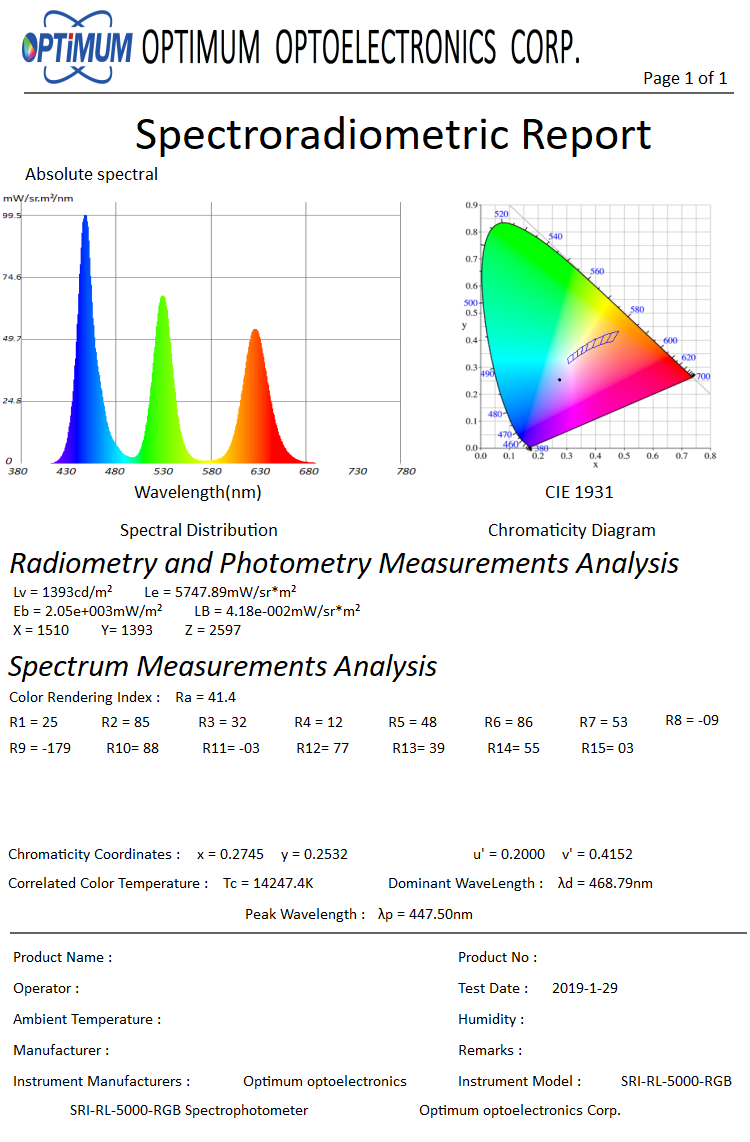


Fig. S12. The L3 spectroradiometric report of 120°-mini-CSPLED with a 150 μm-thick QD films.


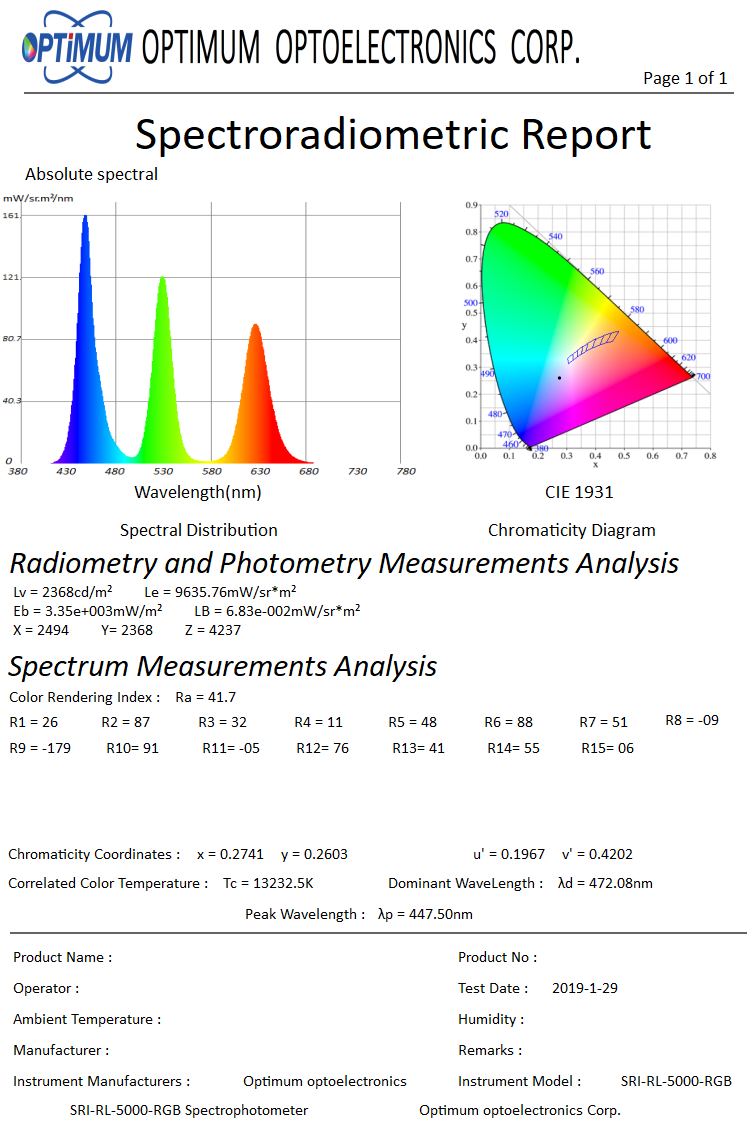


Fig. S13. The L4 spectroradiometric report of 120°-mini-CSPLED with a 150 μm-thick QD films.


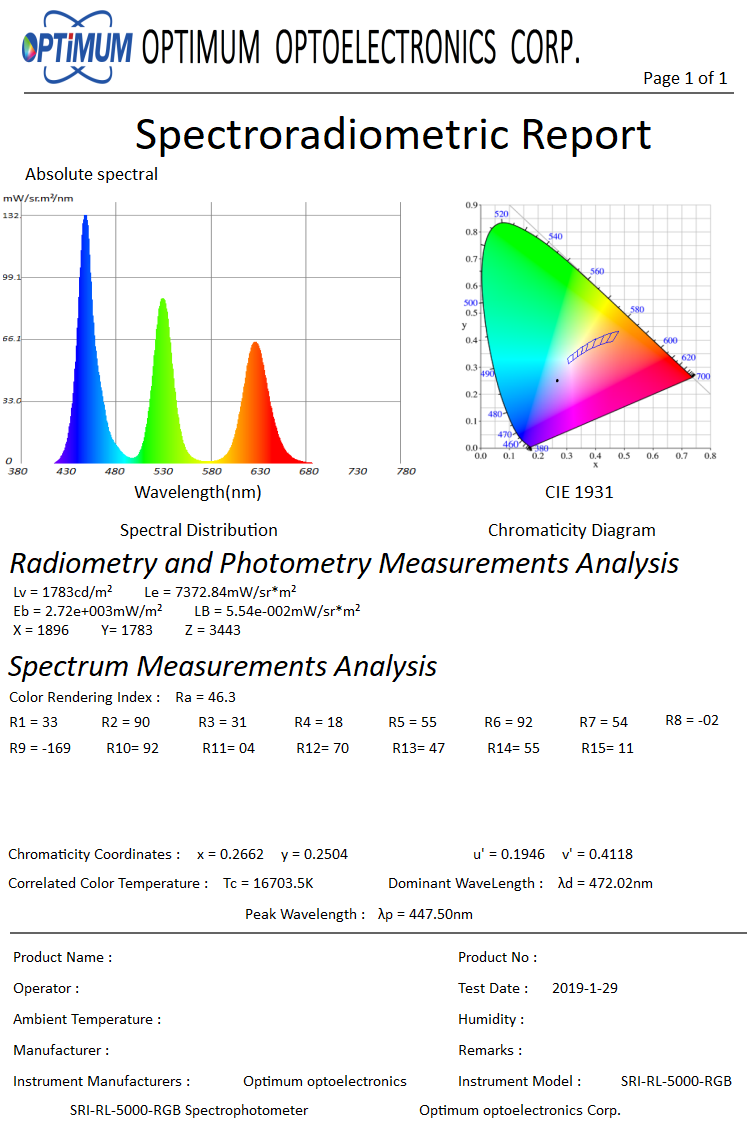


Fig. S14. The L5 spectroradiometric report of 120°-mini-CSPLED with a 150 μm-thick QD films.


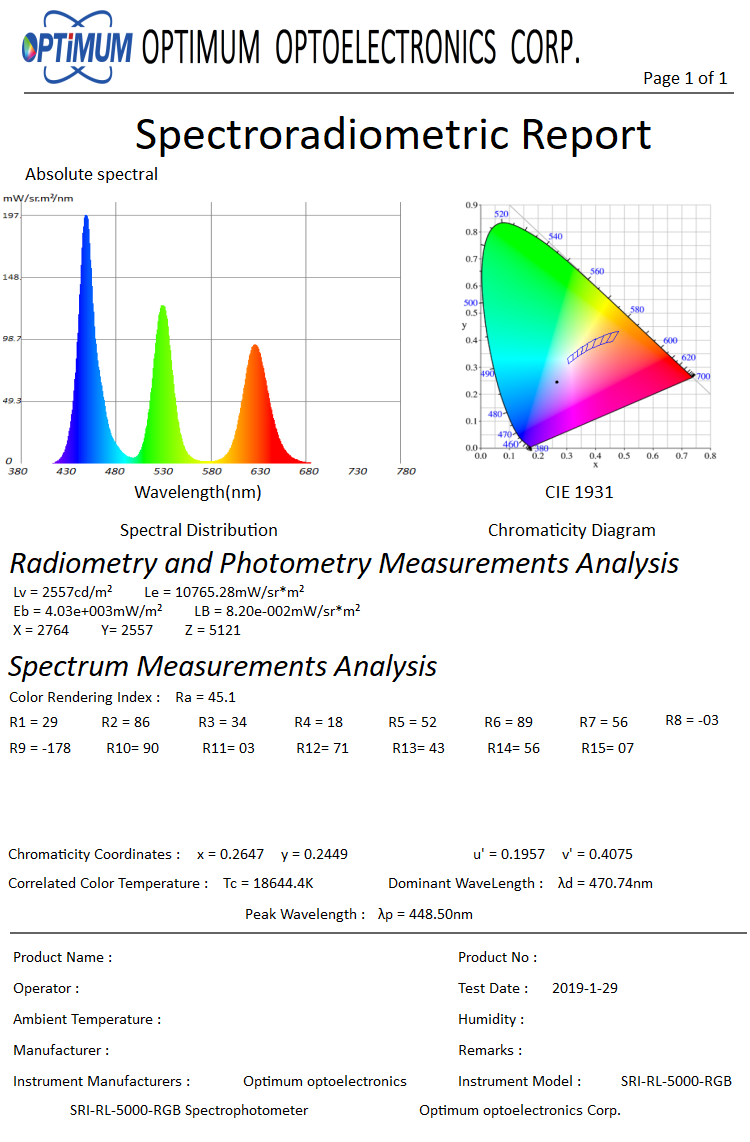


Fig. S15. The L2 spectroradiometric report of 150°-mini-CSPLED with a 150 μm-thick QD films.


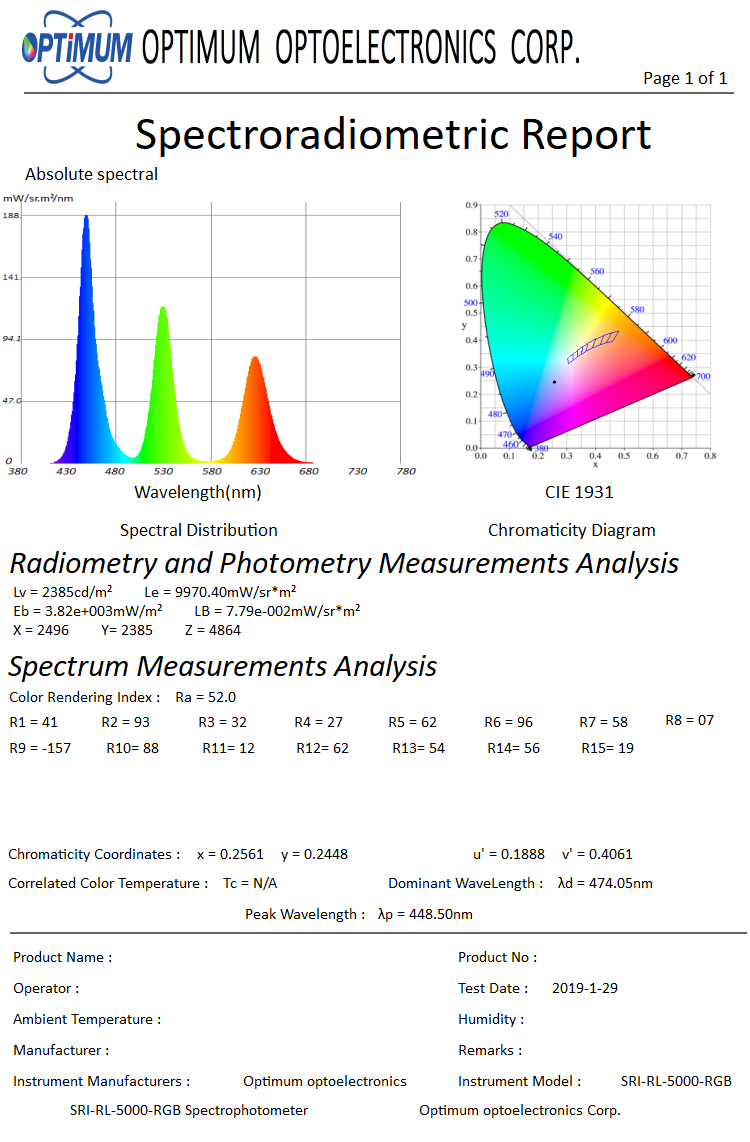


Fig. S16. The L3 spectroradiometric report of 150°-mini-CSPLED with a 150 μm-thick QD films.


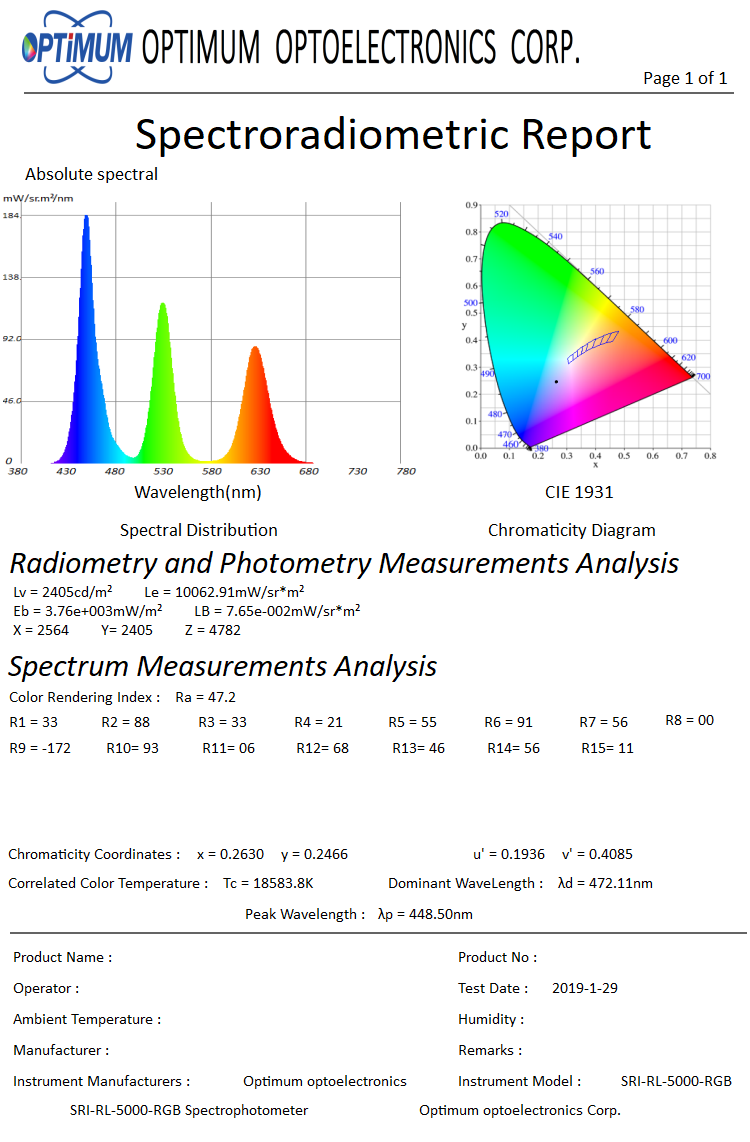


Fig. S17. The L4 spectroradiometric report of 150°-mini-CSPLED with a 150 μm-thick QD films.


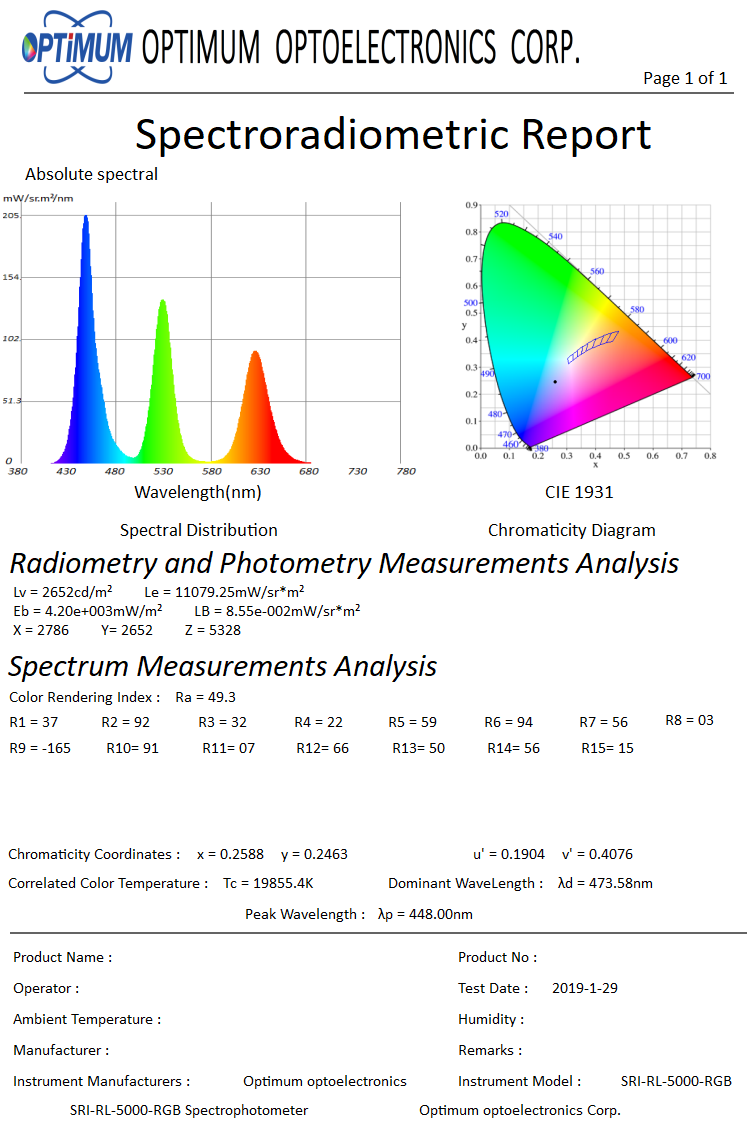


Fig. S18. The L5 spectroradiometric report of 150°-mini-CSPLED with a 150 μm-thick QD films.


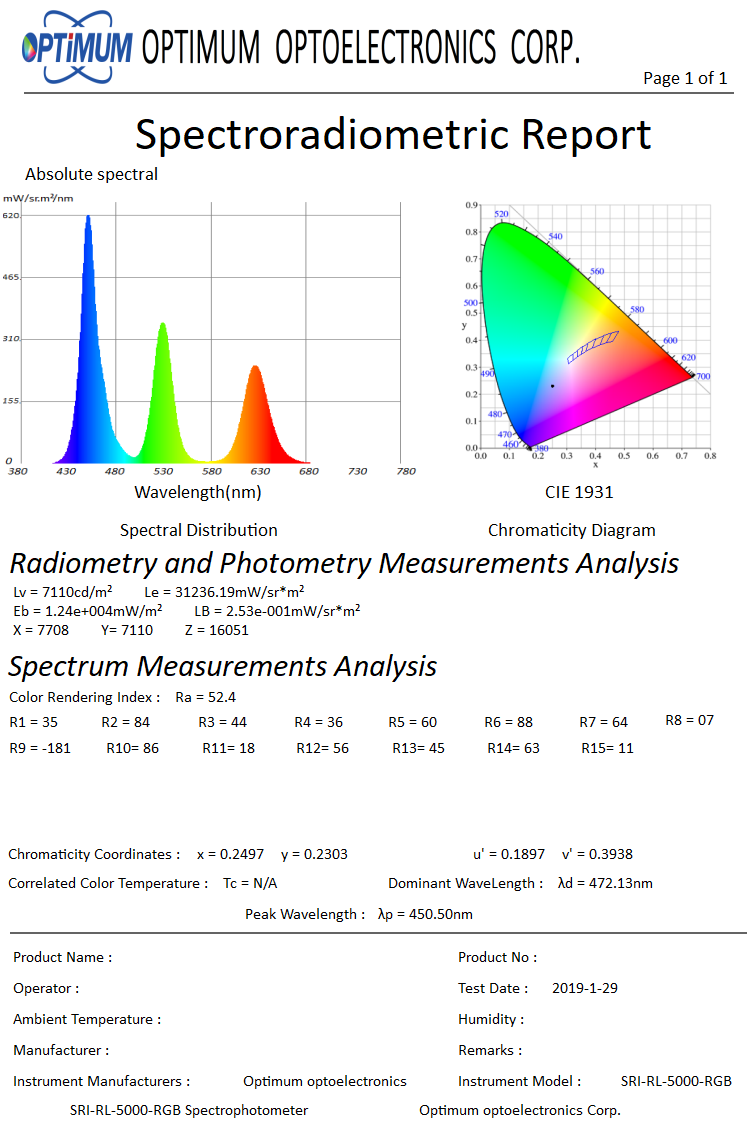


Fig. S19. The L2 spectroradiometric report of 180°-mini-CSPLED with a 150 μm-thick QD films.


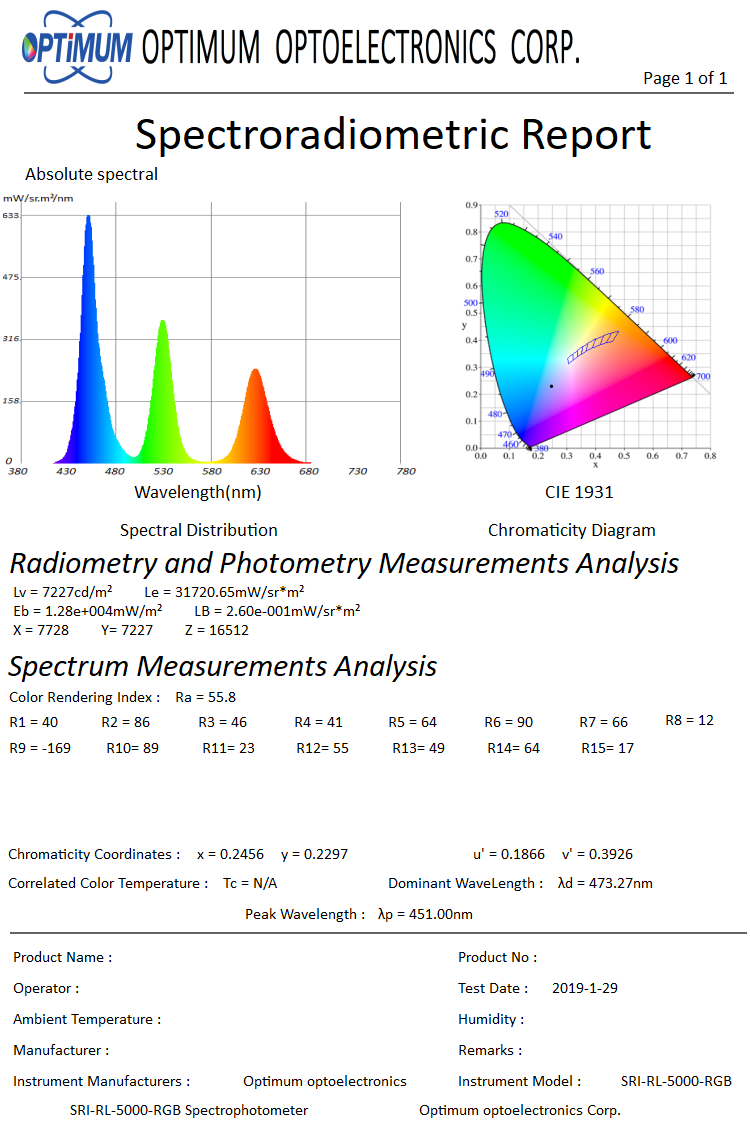


Fig. S20. The L3 spectroradiometric report of 180°-mini-CSPLED with a 150 μm-thick QD films.


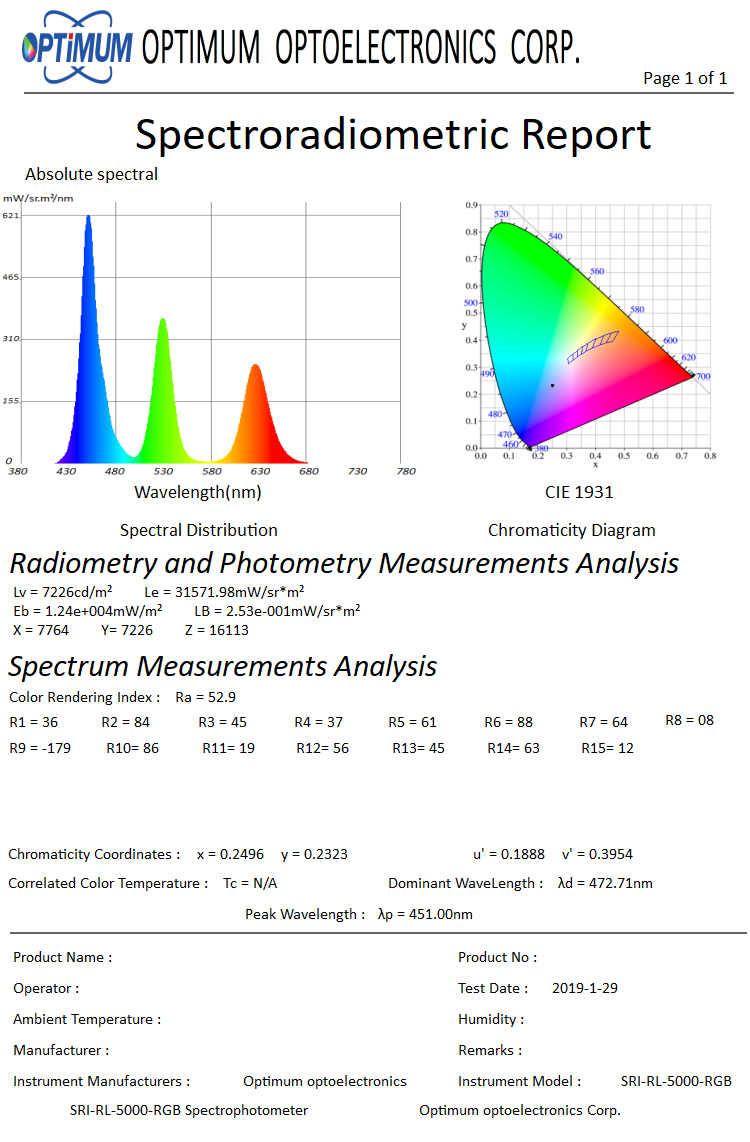


Fig. S21. The L4 spectroradiometric report of 180°-mini-CSPLED with a 150 μm-thick QD films.


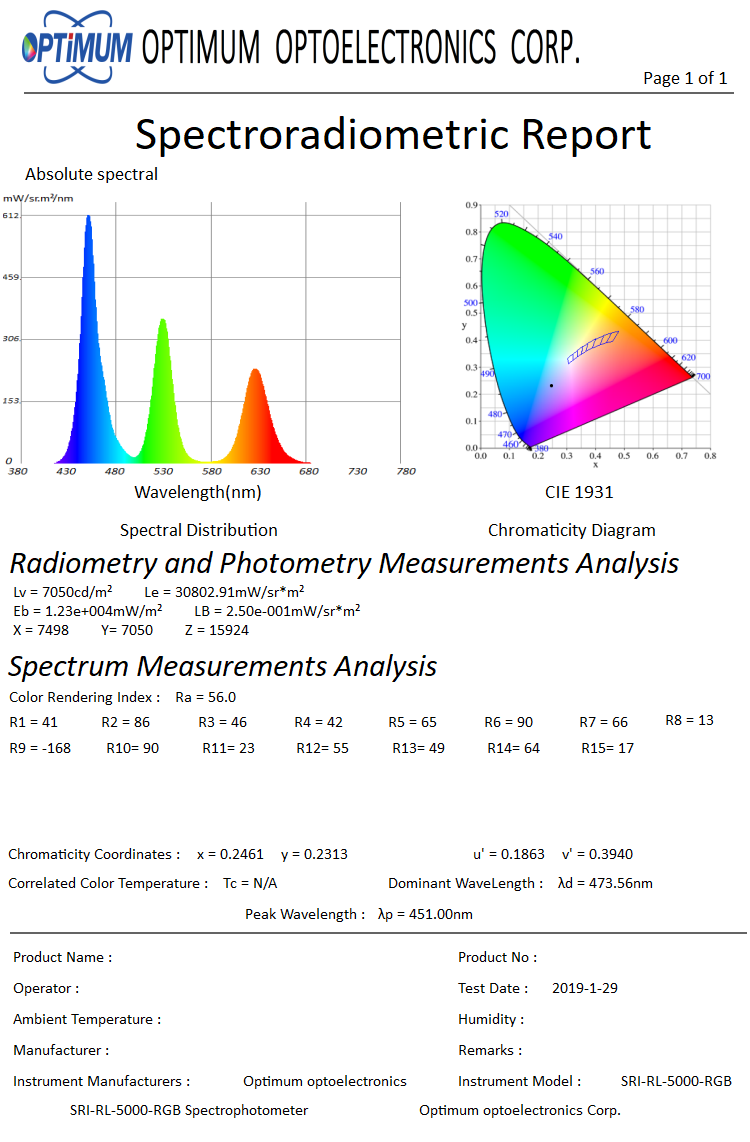


Fig. S22. The L5 spectroradiometric report of 150°-mini-CSPLED with a 150 μm-thick QD films.


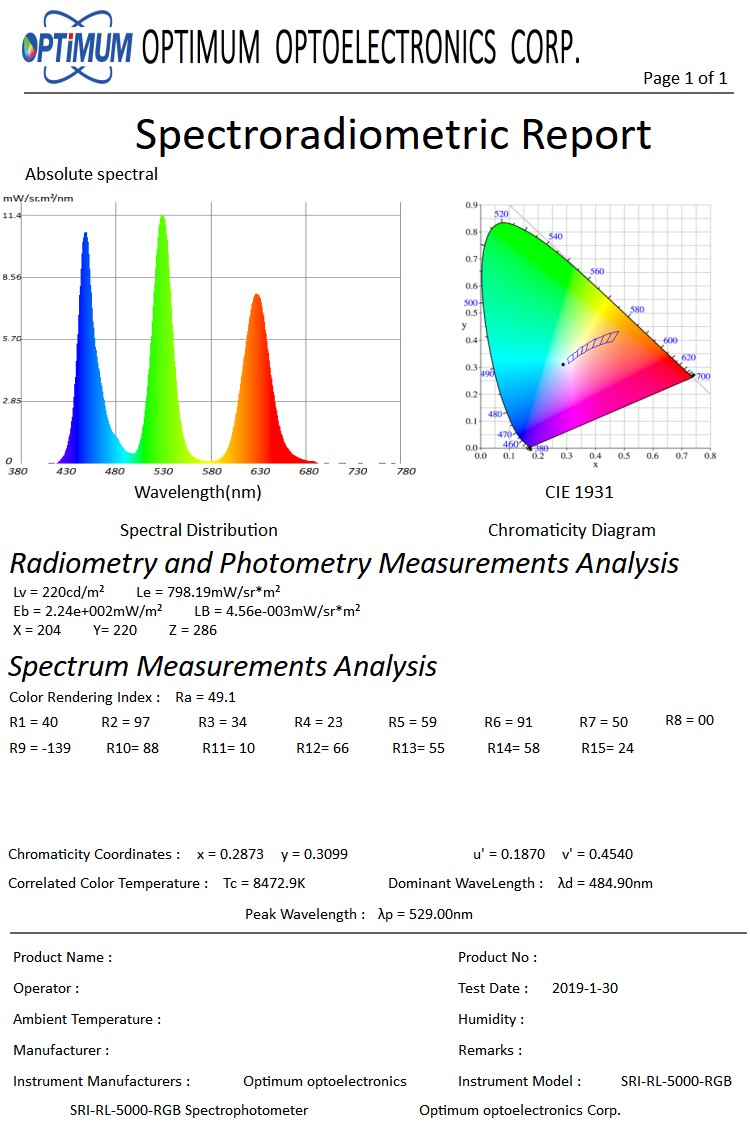


Fig. S23. Spectroradiometric report of 120°-mini-CSPLED + 150 μm-thick QD films with LCD.


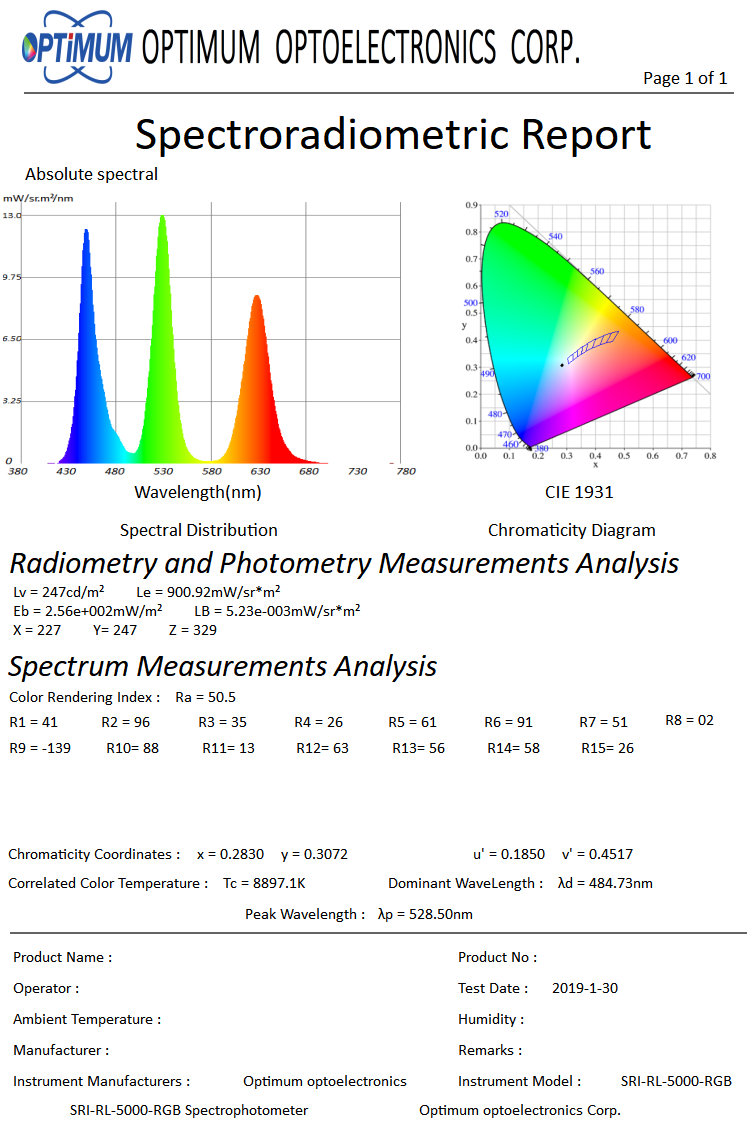


Fig. S24. Spectroradiometric report of 150°-mini-CSPLED + 150 μm-thick QD films with LCD.


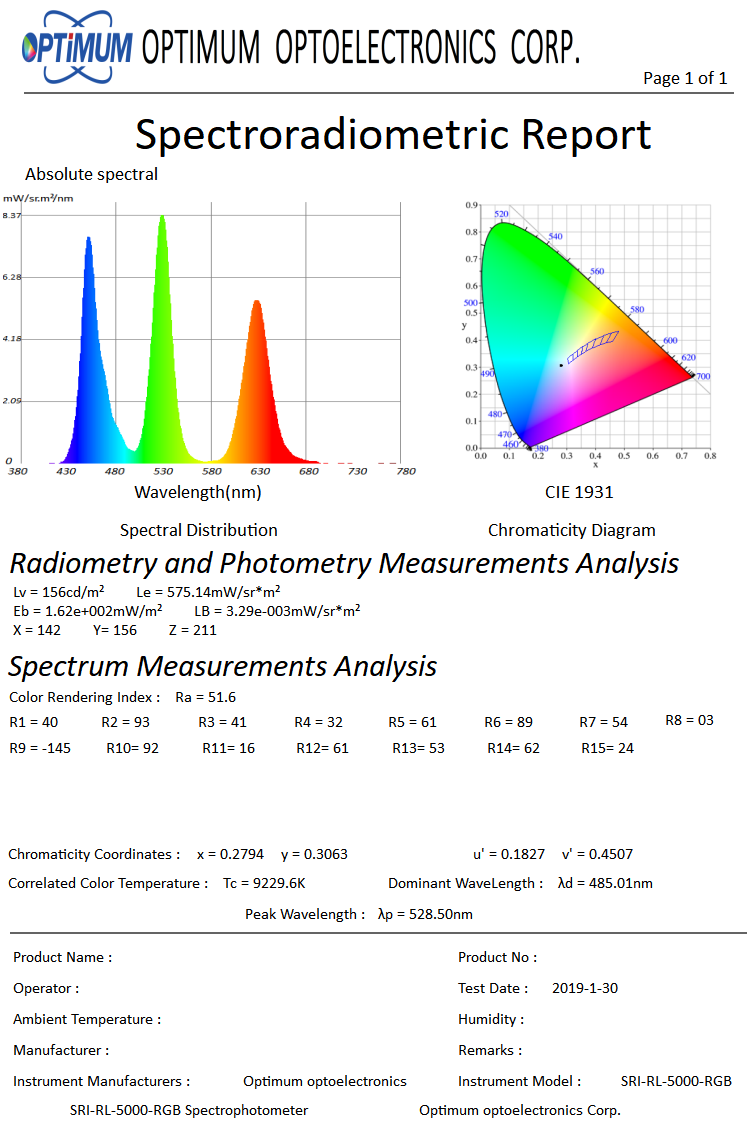


Fig. S25. Spectroradiometric report of 180°-mini-CSPLED + 150 μm-thick QD films with LCD.
